# First total synthesis of (±)-spirobroussonin B via electromediated oxidative coupling for constructing spirodienone ring

**DOI:** 10.1007/s13659-026-00630-2

**Published:** 2026-05-08

**Authors:** Hong-Ji Shi, Xing-Ping Zhang, Cong-Xi Gao, Kun Xie, Wu-Yan Chen, Ming-Ming Li, Kou Wang

**Affiliations:** 1 School of Pharmaceutical Science and Yunnan Key Laboratory of Pharmacology for Natural Products, Yunnan College of Modern Biomedical Industry, Kunming Medical University, Kunming, 650500 China; 2 School of Ethnic Medicine, Yunnan University of Chinese Medicine, Kunming, 650500 China

**Keywords:** Spirobroussonin B, Electromediated, 1,3-Diphenylpropane, Spirodienone fragment

## Abstract

An electromediated oxidative coupling strategy for constructing spirodienone fragment has been developed and applied as the key step for the total synthesis of (±)-spirobroussonin B. This concise route was accomplished in only four steps, 41.6% overall yield and the key coupling reaction was demonstrated on a gram scale. The absolute configurations of the enantiomers were unambiguously determined by chiral resolution and ECD calculations. This work establishes a solid foundation for developing practical and efficient syntheses of spirobroussonin B and related analogues.

**Graphical Abstract:**

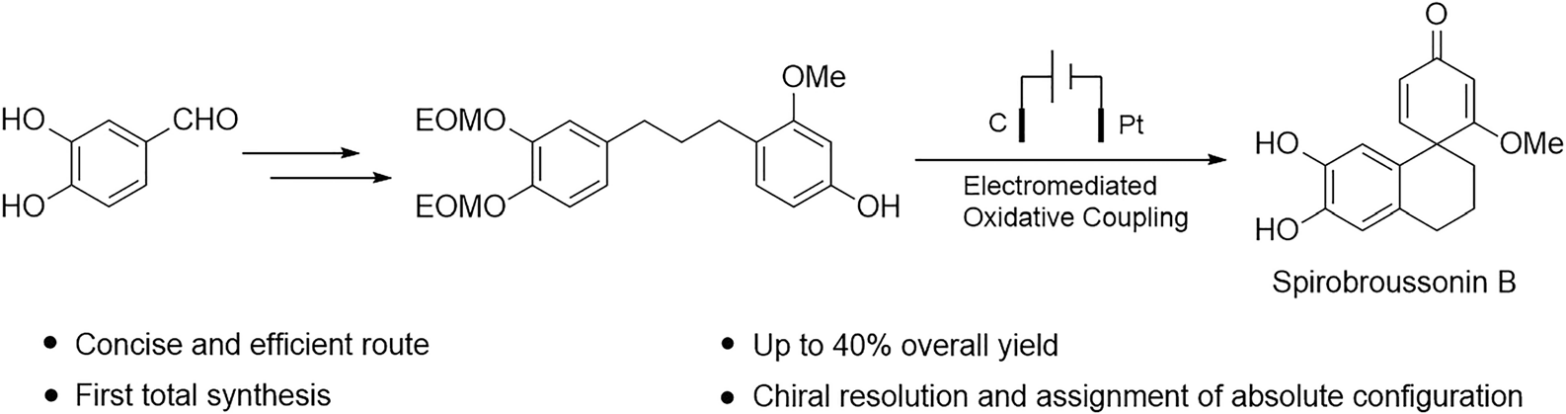

**Supplementary Information:**

The online version contains supplementary material available at 10.1007/s13659-026-00630-2.

## Introduction

**Fig. 1:**
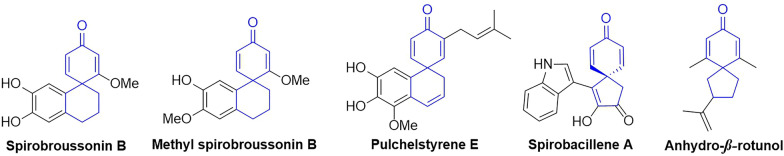
Structures of spirobroussonin B and related natural products

Spirodienone derivatives are characterized by a central spiro atom, a quaternary carbon that bridges a dienone moiety with various four- to seven-membered heterocyclic or carbocyclic rings. This spirodienone motif is a prevalent and critical scaffold in numerous biologically active natural products (Fig. 1) [1–5]. A prominent example is spirobroussonin B, isolated from *Broussonetia papyrifera*, which exhibits notable anti-inflammatory properties [1].

**Scheme 1:**
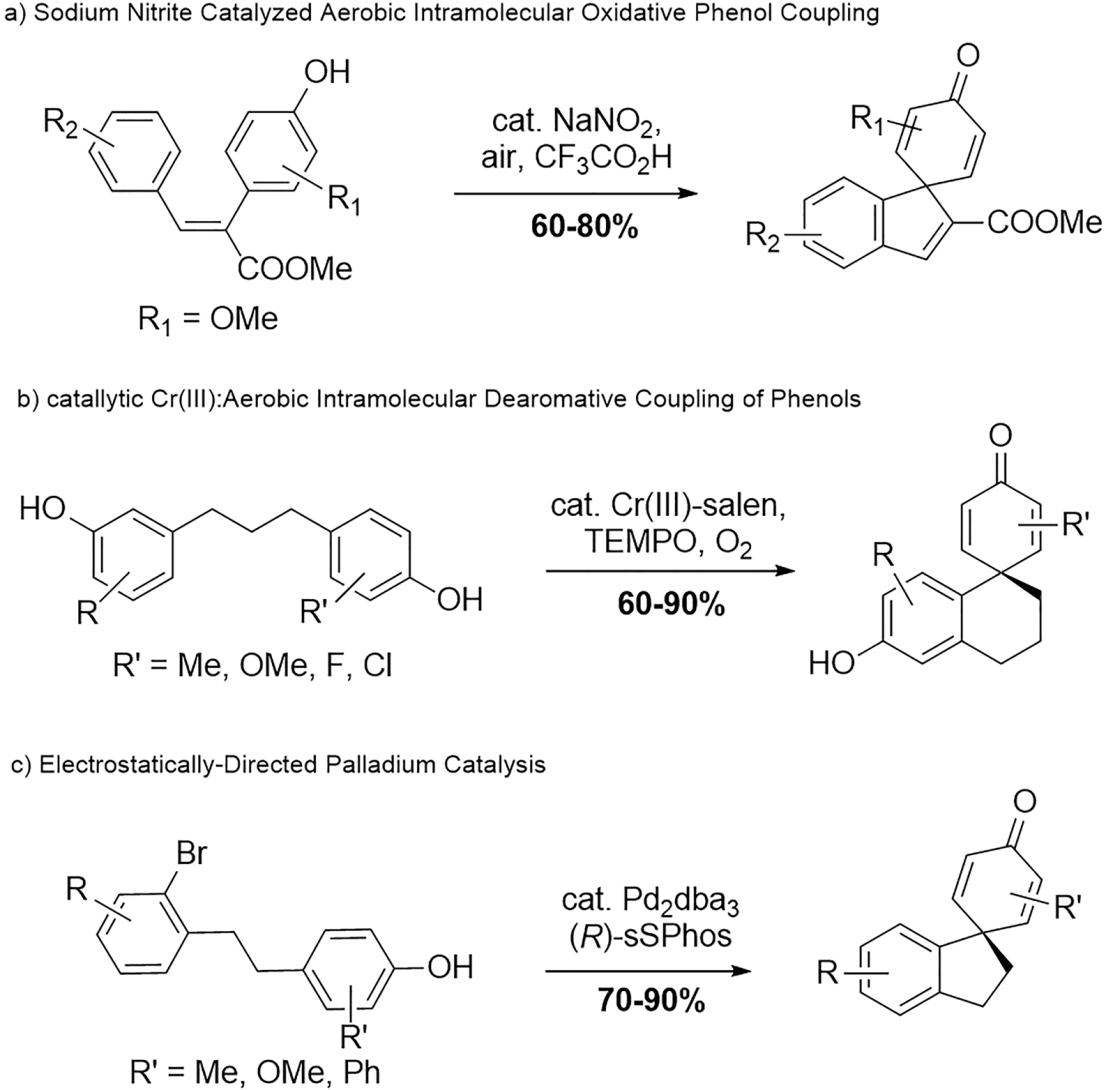
Intramolecular phenol-type couplings

The synthesis of spirodienone derivatives has attracted the extensive interest of researchers, due to the significant activities and small amount obtained from natural sources. Intermolecular oxidative phenol coupling has been extensively explored [6–8], including through transition metal catalysis [9, 10], photocatalysis, and electrocatalysis [11–15]. However, the intramolecular oxidative coupling reaction to form spirodienone has not been fully developed. In 2013, the Wang group reported an approach using sodium nitrite catalyzed oxidative phenol coupling for the construction of the spirocyclohexadienone moiety in the presence of air (Scheme 1a) [16]. More recently, the Iwabuchi group developed a catalytic system based on chromium-salen complexes and nitroxyl radicals, enabling the efficient aerobic intramolecular dearomatization coupling of immobilized phenolic compounds under mild conditions, including the synthesis of products that were previously challenging to obtain (Scheme 1b) [17]. Furthermore, in 2023, the Kadarauch group reported a palladium-catalyzed arylative dearomatization of phenols to yield spirocyclohexadienon, the study also showed that the formation of the spirocyclic all carbon quaternary center can be accomplished with high levels of enantiocontrol (Scheme 1c) [18]. However, these methods rely on the use of acids or transition metals, which can be costly and potentially harmful to the environment [19–23]. In recent years, the advancement of electrochemical synthesis has established it as a compelling strategy in modern organic chemistry. This approach not only minimizes the reliance on transition metal catalysts, aligning with principles of green chemistry, but also offers operational simplicity, reduced risk, and inherent safety due to the avoidance of toxic reagents. Consequently, the development of an electrocatalytic method for oxidative coupling represents a highly desirable and timely objective.

We embarked on a total synthesis of spirobroussonin B, in the hope of finding a new method to construct spirodienone ring. Our synthesis commenced with commercially available 3,4-dihydroxybenzaldehyde as the starting material. This study aims to develop an environmentally sustainable method for the intramolecular dearomatization coupling of phenolic compounds, thereby providing sufficient material for subsequent biological characterization.

### Results and discussion

**Scheme 2:**
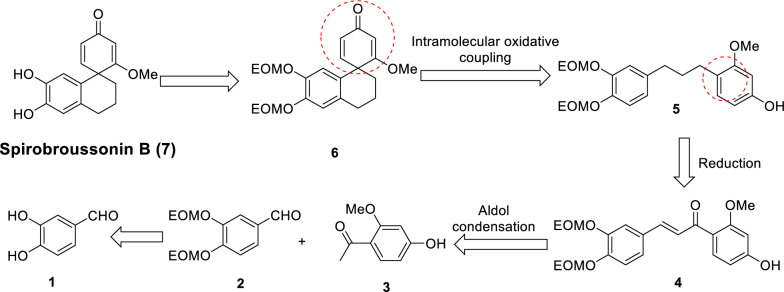
Retrosynthetic analysis of spirobroussonin B

Retrosynthetic analysis revealed that spirobroussonin B (Scheme 2) could be accessed through deprotection of compound **6**. Compound **6** was proposed to be obtained via intramolecular oxidative coupling of 1,3-diphenylpropane** 5**. Compound **5** was yielded from Pd-catalyzed hydrogenation of chalcone **4**. While, compound **4** could be assembled through aldol condensation between 2-methoxyacetophenone **3** and aldehyde **2**, which was obtained from readily available aldehyde **1**.

**Scheme 3:**
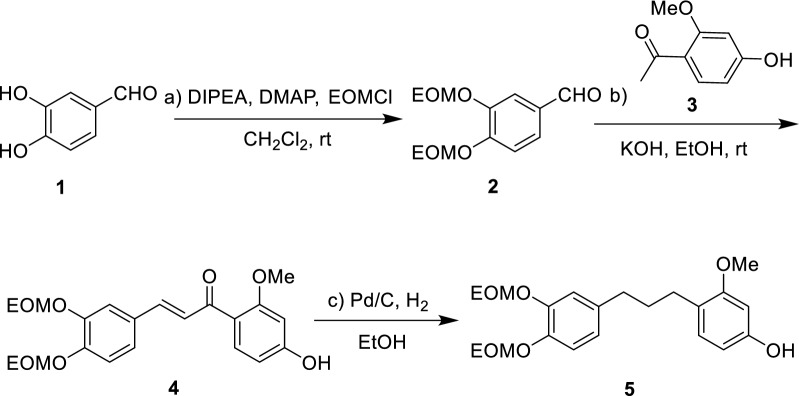
Synthesis of Compound **5**. Reaction conditions for the synthesis of **5**: **a** 1 (1.0 equiv), DMAP (0.1 equiv), DIPEA (4.0 equiv), EOMCl (4.0 equiv), DCM, rt, 7 h; **b** 2 (1.0 equiv), 1-(4-hydroxy-2-methoxyphenyl)ethanone (1.2 equiv), KOH (10.0 equiv), EtOH, rt, 1.5 h; **c** 4 (1.0 equiv), Pd/C (10 mol %), H_2_, EtOH, rt, 48 h

The synthesis commenced with 3,4-dihydroxybenzaldehyde as the starting material (Scheme 3). Treatment with *N*, *N*-diisopropylethylamine (DIPEA), 4-dimethylaminopyridine (DMAP), and chloromethyl ethyl ether (EOMCl) in dichloromethane at room temperature afforded compound **2** in 92.5% yield. Subsequent aldol condensation of compound **2** with 1-(4-hydroxy-2-methoxyphenyl) ethanone **3** under KOH/EtOH conditions furnished compound **4** in 66.8% yield. Reduction of compound **4** via catalytic hydrogenation using Pd/C in EtOH provided compound **5** in 90.6% yield. This transformation was successfully conducted on a gram scale.

To construct the spirocyclohexadienone moiety, we explored the key intramolecular oxidative coupling of compound **5** under electromediated conditions. Initial attempts using Oxone as the oxidant and various electrodes (Table 1, entries 1–2) revealed that both C–Pt and C–C electrodes afforded the desired product **7**, with the C–Pt electrode giving a marginally higher yield. Solvent screening identified MeCN/H₂O (9:1) as optimal (Table 1, entries 2–3). Evaluation of electrolytes (Table 1, entries 3–5) showed that TBAPF₆ performed best, raising the yield of **7** to 41.3%. Further optimization established that 4 equivalents of Oxone and a current of 8 mA provided the most favorable results. Finally, we observed that a portion of the product remained protected. The addition of FeBr₃ (10 mol%) after the reaction enabled complete deprotection and increased the yield to 74.4% (Table 1, entry 10).

**Table 1:** Optimization of the reaction conditions for the synthesis of spirobroussonin B^a^


Entry	Electrodes	Solvent	Electrolyte	Oxone (equiv)	Current(mA)	Yield^b^ (%)
1^c^	C–C	MeOH/H_2_O(9/1)	TBAF	4	10	18.6
2	C–Pt	MeOH/H_2_O(9/1)	TBAF	4	10	20.9
3	C–Pt	MeCN/H_2_O(9/1)	TBAF	4	10	22.1
4	C–Pt	MeCN/H_2_O(9/1)	TBAB	4	10	Trace
5	C–Pt	MeCN/H_2_O(9/1)	TBAPF_6_	4	10	41.3
6	C–Pt	MeCN/H_2_O(9/1)	TBAPF_6_	2	10	18.7
7	C–Pt	MeCN/H_2_O(9/1)	TBAPF_6_	6	10	20.6
8	C–Pt	MeCN/H_2_O(9/1)	TBAPF_6_	4	12	23.0
9	C–Pt	MeCN/H_2_O(9/1)	TBAPF_6_	4	8	53.3
**10**^**d**^	**C**–**Pt**	**MeCN/H**_**2**_**O(9/1)**	**TBAPF**_**6**_	**4**	**8**	**74.4**

^a^Reaction conditions: unless specified otherwise, all reactions were performed with **5** (0.15 mmol), graphite anode and Pt plate cathode (52.5 mm × 8.0 mm × 1.5 mm), solvent (10 ml), electrolyte (2.0 equiv), Oxone (4.0 equiv), constant current, r.t. for 10 h. ^b^Isolated yields, ^c^graphite cathode, ^d^ work-up with FeBr_3_ (10 mol%) for 4 h

**Scheme 4:**
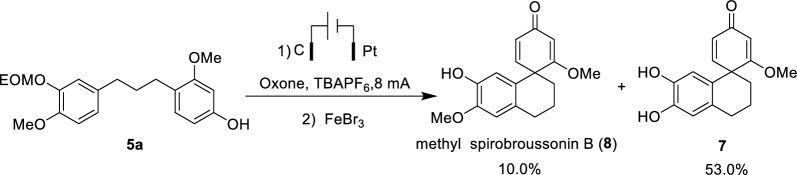
Synthesis of methyl spirobroussonin B

Next, the utility of our methodology was demonstrated. The synthesis of spirobroussonin B in gram-scale reaction was conducted, yielding the final product in 51.1%. When substrate **5a** was subjected to the same conditions, full conversion was observed, yielding methyl spirobroussonin B (**8**) and **7** in 10.0% and 53.0% yield, respectively. It is noted that compound **8** could be further converted into **7** under the reaction conditions (Scheme 4). Subsequent efforts to extend the universality of this method by varying the substituents and their positions on the substrate were unsuccessful, failing to deliver the expected products.

**Scheme 5:**
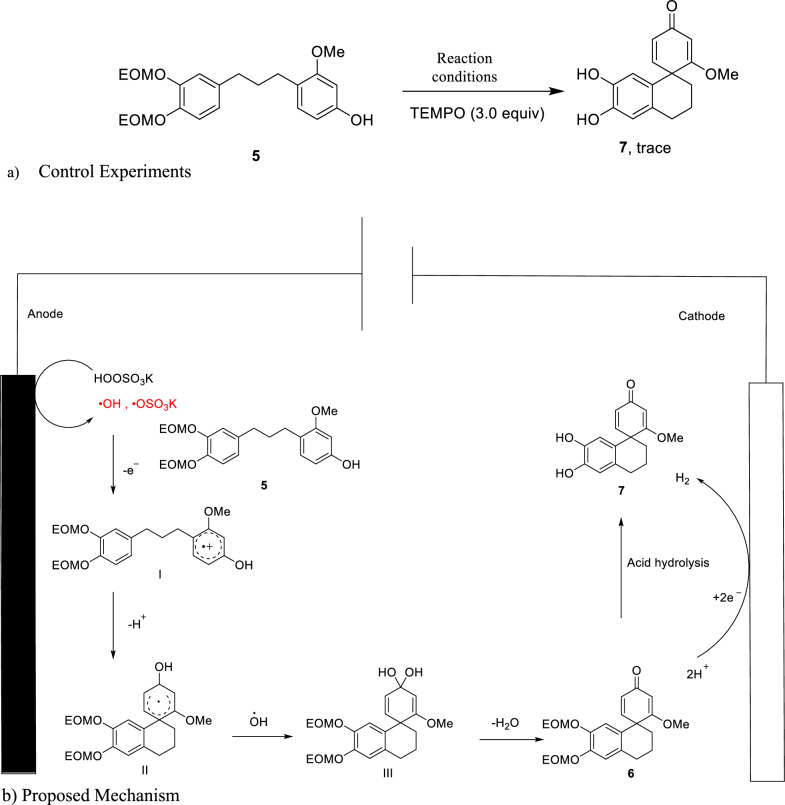
Control experiments and proposed mechanism

Furthermore, the mechanism underlying this key transformation was tested (Scheme 5). In the absence of electromediated, the desired product **7** was not detected, indicating that the reaction was electrically driven. Without the addition of oxone, only a small amount of product was formed, demonstrating the requirement of an external oxidant for the oxidation process. The addition of TEMPO resulted in the detection of an ion peak with a molecular weight of 546 by LC–MS and product **7** trace yield, suggesting that the process was inhibited by TEMPO, implying a potential free radical reaction mechanism (Scheme 5a).

The proposed mechanism [10, 12–14] (Scheme 5b) is as follows: Firstly, compound** 5** was oxidized at the anode in the presence of potassium hydrogen sulfate to form intermediate I. Intermediate I then underwent deprotonation to form intermediate II. Afterward, II reacts with radical •OH to produce the intermediate III. Then, III is converted to the compound **6** by dehydrate. Finally, compound **6** was deprotected by acid hydrolysis to obtain the final product **7**.

**Scheme 6:**
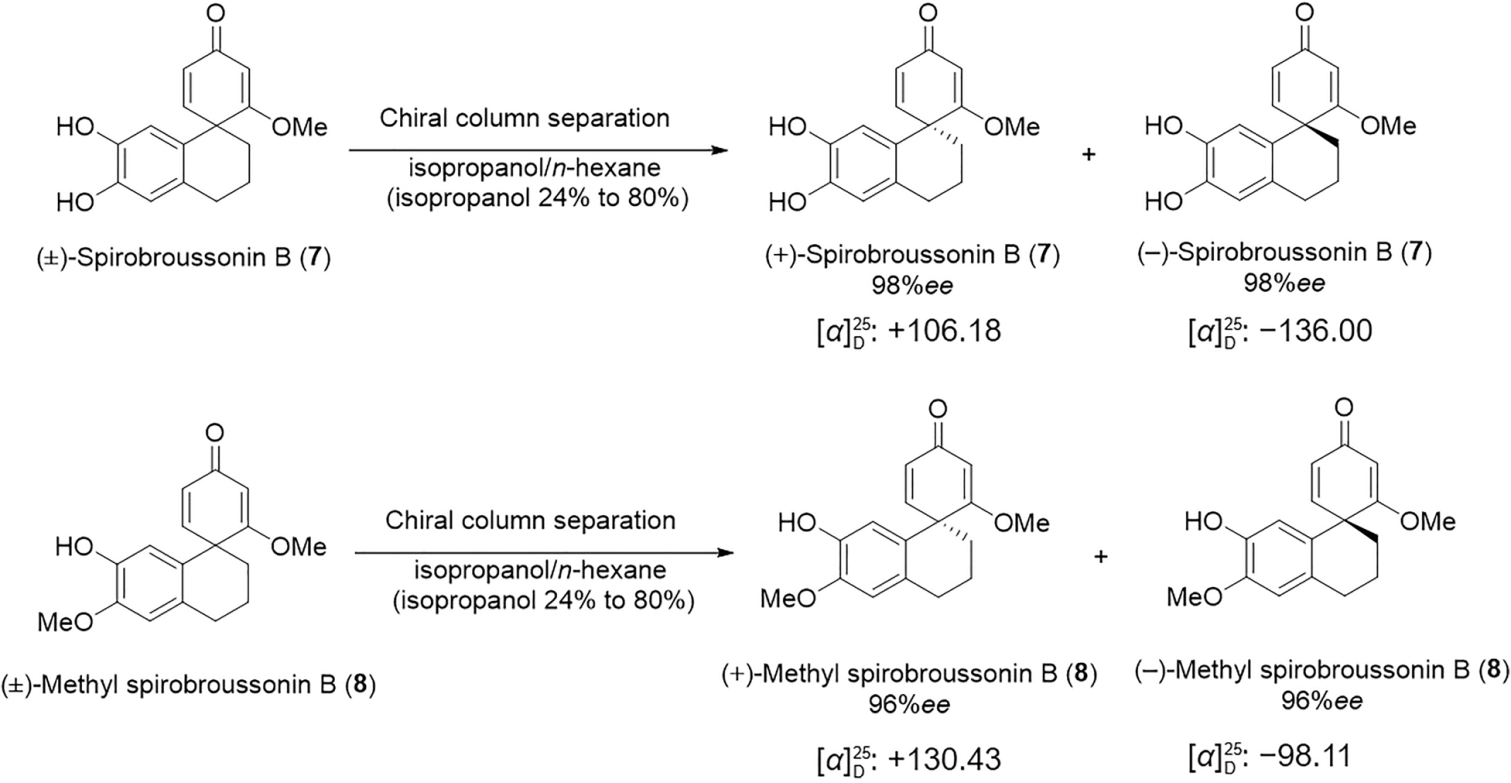
Resolution of enantiomers via separation from **7** and **8**

**Fig. 2:**
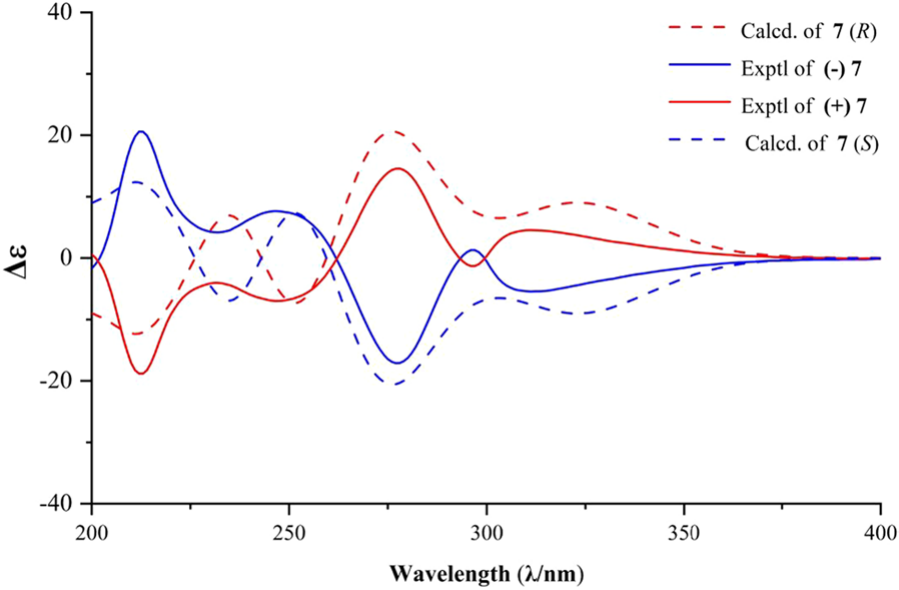
Experimental and calculated ECD spectra of **7**

**Fig. 3:**
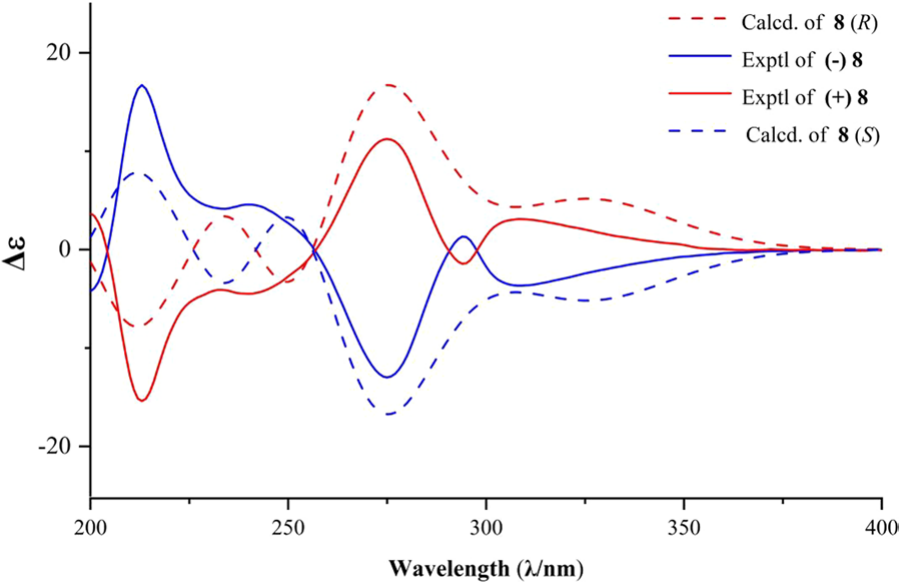
Experimental and calculated ECD spectra of **8**

The synthesized compounds were compared with data reported in the literature [1, 2]. With sufficient amount of compounds **7** and **8** were prepared by our method. To facilitate follow-up pharmacological activity studies and structure–activity relationship (SAR) analysis, we subjected this class of compounds to chiral resolution. This process yielded the separated enantiomers (+)-**7**, (−)-**7**, (+)-**8**, and (−)-**8** (Scheme 6). The absolute configurations of (+)-**7** and (+)-**8** were determined as *R* and *R*, and (−)-**7** and (−)-**8** were determined as *S* and *S* through ECD analysis (Figs. 2, 3).

## Conclusions

In summary, we report the first total synthesis of the natural product spirobroussonin B, accomplished in only four steps in 41.6% overall yield. A key step involves a novel electromediated intramolecular oxidative coupling of a 1, 3-diarylpropane to construct the spirodienone motif. This versatile strategy was also applied to access the related natural product, methyl spirobroussonin B. The absolute configurations of both compounds were unambiguously assigned by chiral separation and ECD calculations. Furthermore, the synthesis of spirobroussonin B was demonstrated on a gram scale. This work provides a robust foundation for further biological studies.

## Experimental procedures

### General experimental protocols

All reactions conducted at temperatures higher than the ambient laboratory temperature were carried out in a silicone oil bath, which was preheated to the target temperature before the reaction vessels were immersed in it. Anhydrous solvents were purified and dried according to standard procedures, while commercially available reagents were used without further purification. Column chromatography was typically performed using silica gel (200–300 mesh, Qingdao Marine Chemical Co. Ltd., Qingdao, People’s Republic of China) as the stationary phase. The progress of the reactions was monitored in real time by thin-layer chromatography (TLC), with the reaction progress observed under ultraviolet light and stained with phosphomolybdic acid in ethanol solution and heat. Petroleum ether: ethyl acetate was used as the eluent. IKA ElectraSyn 2.0 electrolysis devise was used as electrolytic instrument, which was equiped graphite plates electrode (5.2 cm × 0.8 cm × 0.2 cm) as anode and cathode.

Optical rotations were measured using a Jasco P-1020 polarimeter equipped with a 1 dm pathlength cell.^1^H and ^13^C NMR spectra were recorded with tetramethylsilane as an internal standard at ambient temperature with Zhongke 400 MHz or Bruker 600 MHz NMR spectrometer in CDCl_3_ or CD_3_OD unless specified. Splitting patterns are designated as singlet (s), broad singlet (br s), doublet (d), triplet (t), quartet (q), quintet (quin), doublet of doublets (dd), and triplet of doublets (td). Splitting patterns that cannot be interpreted or easily visualized are designated multiplet (m). HRMS data were recorded via positive ion electrospray or electron impact mass spectrometry using a time of flight analyzer. Experimental ECD spectra were measured on a Chirascan instrument. Chiral semi-preparative HPLC was performed on an Agilent 1100 HPLC with the CHIRALPAK^®^ AD-H (4.6 mm × 250 mm) column.

#### Synthesis of 3,4-bis(ethoxymethoxy)benzaldehyde, 2

To a dried reaction flask charged with 3,4-dihydroxybenzaldehyde (1.40 g, 10.0 mmol) in CH_2_Cl_2_ (50 mL) were sequentially added 4-dimethylaminopyridine (122 mg, 1.00 mmol), *N*, *N*-diisopropylethylamine (5.20 g, 40.0 mmol), and chloromethyl ethyl ether (3.80 g, 40.0 mmol). The flask was fitted with a drying tube, and the reaction mixture was stirred at room temperature for 7 h. Upon completion of the reaction as monitored by TLC (disappearance of the starting material), the mixture was quenched with water and extracted with CH_2_Cl_2_. The combined organic layers were dried over anhydrous Na_2_SO_4_ and concentrated under reduced pressure. Purification by silica gel column chromatography (petroleum ether/ethyl acetate, 15:1, v/v) afforded compound **2** as a yellow–brown oil (2.35 g, 92.5% yield). ^1^H NMR (600 MHz, CDCl_3_) *δ*: 9.86 (s, 1H), 7.68 (d, *J* = 2.0 Hz, 1H), 7.50 (dd, *J* = 8.3, 2.0 Hz, 1H), 7.30 (d, *J* = 8.4 Hz, 1H), 5.36 (s, 2H), 5.32 (s, 2H), 3.80–3.74 (m, 5H), 1.22 (td, *J* = 7.1, 3.8 Hz, 7H). ^13^C NMR (150 MHz, CDCl_3_) *δ*: 191.0, 152.9, 147.6, 131.1, 126.3, 116.0, 115.4, 94.1, 93.7, 64.9, 64.8, 15.2, 15.1. HRMS (ESI) *m/z*: [M + H]^+^ calcd. for [C_13_H_18_O_5_ + H]^+^ 255.2901, found: 255.1235.

#### Synthesis of (E)-3-[3,4-bis(ethoxymethoxy)phenyl]-1-(4-hydroxy-2-methoxyphenyl)prop-2-en-1-one, 4

Compound **2** (1.27 g, 5.0 mmol) was dissolved in EtOH (40 mL) in a dried reaction flask, followed by the addition of 1-(4-hydroxy-2-methoxyphenyl)ethan-1-one (830 mg, 5.0 mmol) and potassium hydroxide (2.8 g, 50 mmol). The reaction mixture was stirred at room temperature for 1.5 h, at which point TLC analysis indicated the almost complete consumption of the starting material. The reaction was then quenched by adjusting the pH to neutrality and adding water. The resulting mixture was concentrated under reduced pressure to remove EtOH, followed by extraction with CH_2_Cl_2_. The combined organic layers were dried over anhydrous Na_2_SO_4_ and concentrated under reduced pressure to afford the crude product. Purification by silica gel column chromatography (petroleum ether/ethyl acetate, 3:1, *v*/*v*) furnished compound **4** as a yellow oil (1.34 g, 66.8% yield). ^1^H NMR (600 MHz, CD_3_OD) *δ*: 7.62 (d, *J* = 8.5 Hz, 1H), 7.54 (d, *J* = 15.7 Hz, 1H), 7.50 (d, *J* = 15.7 Hz, 1H), 7.47 (d, *J* = 2.1 Hz, 1H), 7.25 (dd, *J* = 8.4, 2.1 Hz, 1H), 7.18 (d, *J* = 8.4 Hz, 1H), 6.52 (d, *J* = 2.1 Hz, 1H), 6.47 (dd, *J* = 8.5, 2.2 Hz, 1H), 5.29 (s, 4H), 3.91 (s, 3H), 3.81–3.73 (m, 4H), 1.22 (dt, *J* = 16.0, 7.1 Hz, 6H). ^13^C NMR (150 MHz, CD_3_OD) *δ*: 192.5, 164.8, 162.7, 150.8, 148.9, 143.0, 133.9, 131.0, 126.9, 124.8, 121.5, 117.8, 117.3, 109.0, 100.1, 95.4, 95.0, 65.6, 65.5, 56.1, 15.5, 15.4. HRMS (ESI) *m/z*: [M + H]^+^ calcd. for [C_22_H_26_O_7_ + H]^+^ 403.4511, found: 403.1760.

#### Synthesis of 4-{3-[3,4-bis(ethoxymethoxy)phenyl]propyl}-3-methoxyphenol, 5

Compound **4** (1.2 g, 3.0 mmol) was dissolved in EtOH (40 mL) in a dried flask containing palladium on carbon (120 mg). The mixture was stirred under a hydrogen atmosphere at room temperature for 48 h, during which the reaction was monitored by TLC. Upon complete consumption of the starting material, the catalyst was removed by filtration, and the filtrate was concentrated under reduced pressure to afford the crude product. Purification by silica gel column chromatography (petroleum ether/ethyl acetate, 5:1, *v*/*v*) furnished compound **5** as a colorless oil (1.06 g, 90.6% yield). ^1^H NMR (600 MHz, CD_3_OD) *δ*: 7.01 (d, *J* = 9.7 Hz, 1H), 6.95 (s, 1H), 6.87 (d, *J* = 8.1 Hz, 1H), 6.76 (d, *J* = 8.2 Hz, 1H), 6.38 (s, 1H), 6.29 (d, *J* = 8.1 Hz, 1H), 5.20 (s, 2H), 5.17 (s, 2H), 3.78–3.73 (m, 7H), 2.51 (q, *J* = 8.3 Hz, 4H), 1.79 (dt, *J* = 14.5, 7.7 Hz, 2H), 1.20 (t, *J* = 7.1 Hz, 6H). ^13^C NMR (150 MHz, CD_3_OD)* δ*: 159.7, 157.7, 148.7, 146.7, 138.8, 131.2, 123.4, 122.6, 118.9, 118.8, 107.5, 99.7, 95.6, 95.4, 65.3, 65.2, 55.6, 36.0, 33.2, 30.2, 15.5, 15.4. HRMS (ESI) *m/z*: [M + Na]^+^ calcd. for [C_22_H_30_O_6_ + Na]^+^ 413.4651, found: 413.1941.

#### Synthesis of 6ʹ,7'-dihydroxy-2-methoxy-3',4'-dihydro-2'H-spiro(cyclohexane-1,1'-naphthalene)-2,5-dien-4-one, 7

Compound **5** (59 mg, 0.15 mmol) was placed in a dry reaction flask equipped with acetonitrile (9 mL) and water (1 mL). To this solution were sequentially added potassium hydrogen persulfate (184 mg, 0.6 mmol) and tetrabutylammonium hexafluorophosphate (116 mg, 0.3 mmol). The electrocatalytic reaction was then carried out using a C-Pt electrode pair at a constant current of 8 mA at room temperature for 4 h. The progress of the reaction was monitored by TLC until the starting material was completely consumed. Upon completion, the electrolysis was stopped, and the mixture was transferred to a clean round-bottom flask, where FeBr_3_ (6 mg) was added and the mixture was stirred for an additional 4 h. Acetonitrile was removed under reduced pressure, and the residue was extracted with ethyl acetate. The combined organic layers were dried over anhydrous Na_2_SO_4_ and concentrated under reduced pressure to give the crude product. Purification by silica gel column chromatography (petroleum ether/ethyl acetate, 2:1, *v*/*v*) afforded compound **7** as a red-brown powder (30.4 mg, 74.4% yield). ^1^H NMR (600 MHz, CD_3_OD) *δ*: 6.94 (d, *J* = 9.8 Hz, 1H), 6.53 (s, 1H), 6.21 (s, 1H), 6.04 (dd, *J* = 9.8, 1.6 Hz, 1H), 5.70 (d, *J* = 1.6 Hz, 1H), 3.69 (s, 3H), 2.75–2.63 (m, 2H), 2.28–2.20 (m, 1H), 2.03–1.77 (m, 3H). ^13^C NMR (150 MHz, CD_3_OD) *δ*: 191.1, 184.0, 154.8, 145.8, 145.0, 130.0, 125.2, 123.7, 117.0, 114.6, 102.5, 56.5, 48.2, 34.7, 29.8, 20.6. HRMS (ESI) *m/z*: [M + H]^+^ calcd. for [C_16_H_16_O_4_ + H]^+^ 273.3079, found: 273.1120. (+)-Spirobroussonin B $[\alpha]^{25}_\mathrm{D}$: + 106.18; (−)-Spirobroussonin B $[\alpha]^{25}_\mathrm{D}$: − 136.00.

#### Synthesis of 4-(3-(3-(ethoxymethoxy)-4-methoxyphenyl)propyl)-3-methoxyphenol, 5a

To a solution of (*E*)-3-(3-(ethoxymethoxy)-4-methoxyphenyl)-1-(4-hydroxy-2-methoxyphenyl)prop-2-en-1-one (1.08 g, 3.0 mmol) in EtOH (40 mL) was added 10% palladium on carbon (120 mg). The mixture was stirred under a hydrogen atmosphere at room temperature for 48 h, and the reaction was monitored by TLC until complete consumption of the starting material. The catalyst was removed by filtration, and the filtrate was concentrated under reduced pressure to give the crude product. Purification by silica gel column chromatography (petroleum ether/ethyl acetate, 5:1, *v*/) afforded compound **5a** as a colorless oil (809.6 mg, 78.0% yield). ^1^H NMR (400 MHz, CD_3_OD) *δ*: 6.92 (d, *J* = 2.1 Hz, 1H), 6.86 (d, *J* = 8.1 Hz, 2H), 6.78 (dd, *J* = 8.2, 2.1 Hz, 1H), 6.38 (d, *J* = 2.4 Hz, 1H), 6.28 (dd, *J* = 8.1, 2.4 Hz, 1H), 5.17 (s, 2H), 3.80 – 3.72 (m, 8H), 2.51 (td, *J* = 7.7, 5.0 Hz, 4H), 1.85 – 1.73 (m, 2H), 1.19 (t, *J* = 7.1 Hz, 3H). ^13^C NMR (100 MHz, CD_3_OD) *δ*: 159.6, 157.7, 149.5, 147.4, 136.9, 131.1, 123.5, 122.6, 118.9, 113.5, 107.5, 99.7, 95.5, 65.3, 56.5, 55.6, 35.9, 33.2, 30.2, 15.4.

#### Synthesis of 7'-hydroxy-2,6'-dimethoxy-3',4'-dihydro-2'H-spiro(cyclohexane-1,1'-naphthalene)-2,5-dien-4-one, 8

Compound **5a** (52 mg, 0.15 mmol) was placed in a dry reaction flask equipped with acetonitrile (9 mL) and water (1 mL). To this solution were sequentially added potassium hydrogen persulfate (184 mg, 0.6 mmol) and tetrabutylammonium hexafluorophosphate (116 mg, 0.3 mmol). The electrocatalytic reaction was then carried out using a C–Pt electrode pair at a constant current of 8 mA at room temperature for 1 h. The progress of the reaction was monitored by TLC until the starting material was completely consumed. Upon completion, the electrolysis was stopped, and the mixture was transferred to a clean round-bottom flask, where FeBr_3_ (6 mg) was added and the mixture was stirred for an additional 4 h. Acetonitrile was removed under reduced pressure, and the residue was extracted with ethyl acetate. The combined organic layers were dried over anhydrous Na_2_SO_4_ and concentrated under reduced pressure to give the crude product. Purification by silica gel column chromatography (petroleum ether/ethyl acetate, 2:1, *v*/*v*) afforded compound **7** as a red-brown powder (21.6 mg, 53% yield) and compound **8** as a white solid (4.3 mg, 10% yield). ^1^H NMR (600 MHz, CD_3_OD) *δ*: 6.98 (d, *J* = 9.8 Hz, 1H), 6.69 (s, 1H), 6.24 (s, 1H), 6.08 (dd, *J* = 9.8, 1.6 Hz, 1H), 5.73 (d, *J* = 1.6 Hz, 1H), 3.83 (s, 3H), 3.72 (s, 3H), 2.79 (dt, *J* = 12.2, 5.6 Hz, 2H), 2.28 (ddd, *J* = 13.8, 11.3, 3.2 Hz, 1H), 2.04–1.83 (m, 3H). ^13^C NMR (150 MHz, CD_3_OD) *δ*: 191.0, 183.7, 154.5, 148.5, 146.1, 130.0, 126.6, 124.0, 114.5, 113.6, 102.6, 56.5, 56.3, 48.2, 34.7, 30.0, 20.6. HRMS (ESI) *m/z*: [M + H]^+^ calcd. for [C_17_H_18_O_4_ + H]^+^ 287.3347, found: 287.1287. (+)-Methyl spirobroussonin B $[\alpha]^{25}_\mathrm{D}$: + 130.43; (−)-Methyl spirobroussonin B $[\alpha]^{25}_\mathrm{D}$: −98.11.

#### Gram-scale synthesis of spirobroussonin B, via electrocatalytic oxidative coupling

Compound **5** (1.10 g, 2.82 mmol) was placed in a dry reaction flask equipped with acetonitrile (36 mL) and water (4 mL). To this solution were sequentially added potassium hydrogen persulfate (3.46 g, 11.3 mmol) and tetrabutylammonium hexafluorophosphate (2.18 g, 5.6 mmol). The electrocatalytic reaction was then carried out using a C-Pt electrode pair at a constant current of 8 mA at room temperature for 6 h. The progress of the reaction was monitored by TLC until the starting material was completely consumed. Upon completion, the electrolysis was stopped, and the mixture was transferred to a clean round-bottom flask, where FeBr_3_ (110 mg) was added and the mixture was stirred for an additional 4 h. Acetonitrile was removed under reduced pressure, and the residue was extracted with ethyl acetate. The combined organic layers were dried over anhydrous Na_2_SO_4_ and concentrated under reduced pressure to give the crude product. Purification by silica gel column chromatography (petroleum ether/ethyl acetate, 2:1, *v*/*v*) afforded compound **7** as a white amorphous powder (392 mg, 51.1% yield).

## Supplementary Information

**Additional file 1.**

## Data Availability

The datasets used or analyzed during the current study are available from the corresponding author on reasonable request.
