# Supplementary material for: First total synthesis of (±)-spirobroussonin B via electromediated oxidative coupling for constructing spirodienone ring
Source: Nat Prod Bioprospect. 2026 May 8;16(1):62. doi: 10.1007/s13659-026-00630-2 (PMC13153339; doi:10.1007/s13659-026-00630-2)
Supplement: Supplementary file 1 — Additional file 1. [file 13659_2026_630_MOESM1_ESM.pdf]

Supporting Information

First Total Synthesis of (±)-Spirobroussonin B via Electromediated Oxidative Coupling for Constructing Spirodienone Ring

Hong-Ji Shi[1], Xing-Ping Zhang[1], Cong-Xi Gao[1], Kun Xie[2], Wu-Yan Chen[1], Ming-Ming Li[2*], Kou Wang[1*]

[1] *School of Pharmaceutical Science and Yunnan Key Laboratory of Pharmacology for Natural Products, Kunming Medical University, Kunming, Yunnan 650500, China*

[2] *School of Ethnic Medicine, Yunnan University of Chinese Medicine, Kunming 650500, China*

* Corresponding authors.

E-mail address: lmm_xiaobai@126.com (M.-m. Li.), wangkou@kmmu.edu.cn (K.Wang).

Table of Contents

I. General Experimental Protocols S2
II. Control Experiments and Proposed Mechanism S3
III. Enantioseparation 7, 8 and Computational details S5
IV. Copies of ^{1}H, ^{13}C NMR Spectra, IR and HRMS S28

I. General Experimental Protocols:

All reactions conducted at temperatures higher than the ambient laboratory temperature were carried out in a silicone oil bath, which was preheated to the target temperature before the reaction vessels were immersed in it. Anhydrous solvents were purified and dried according to standard procedures, while commercially available reagents were used without further purification. Column chromatography was typically performed using silica gel (200-300 mesh, Qingdao Marine Chemical Co. Ltd., Qingdao, People's Republic of China) as the stationary phase. The progress of the reactions was monitored in real time by thin-layer chromatography (TLC), with the reaction progress observed under ultraviolet light and stained with phosphomolybdic acid in ethanol solution and heat. Petroleum ether: ethyl acetate was used as the eluent.IKA ElectraSyn 2.0 electrolysis devise was used as electrolytic instrument, which was equiped graphite plates electrode (5.2cm×0.8cm×0.2cm) as anode and cathode.

Optical rotations were measured using a Jasco P-1020 polarimeter equipped with a 1 dm pathlength cell.^{1}H and ^{13}C NMR spectra were recorded with tetramethylsilane as an internal standard at ambient temperature with Zhongke 400 MHz or Bruker 600 MHz NMR spectrometer in $CDCl_3$ or CD_3OD unless specified. Splitting patterns are designated as singlet (s), broad singlet (br s), doublet (d), triplet (t), quartet (q), quintet (quin), doublet of doublets (dd), and triplet of doublets (td). Splitting patterns that cannot be interpreted or easily visualized are designated multiplet (m). HR-MS data were recorded via positive ion electrospray or electron impact mass spectrometry using a time of flight analyzer. Experimental ECD spectra were measured on a Chirascan instrument. Chiral semi-preparative HPLC was performed on an Agilent 1100 HPLC with the CHIRALPAK® AD-H (4.6 mm × 250 mm) column.

II. Control Experiments and Proposed Mechanism

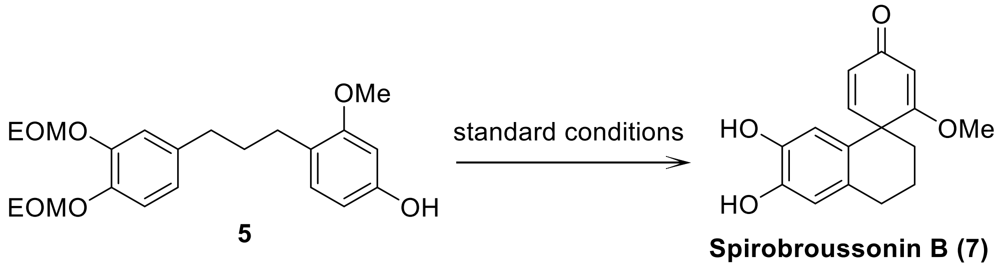

Modified conditions	Results
No electricity	**7** (no detected)
Without Oxone	**7** (trace)
TEMPO (3.0 equiv) was added	**7** (trace） [m/z=546.3443] detected

Under standard experimental conditions, the reaction yields no final product in the absence of an electric current, confirming its electrocatalytic nature. Without Oxone, only minimal product formation occurs. Electron scavengers TEMPO and BHT (uncaught) are then introduced to capture potential intermediates.

Compound 5 (59 mg, 0.15 mmol) was placed in a dry reaction flask and dissolved in acetonitrile (9 mL) with the addition of water (1 mL). Potassium peroxymonosulfate (184 mg, 0.6 mmol), tetrabutylammonium hexafluorophosphate (116 mg, 0.3 mmol), and TEMPO (70.2 mg, 0.45 mmol) were sequentially added. The electrocatalytic reaction was conducted at room temperature using a C-Pt electrode with a constant current of 8 mA for 4 hours. Thin-layer chromatography was employed to monitor the reaction until the starting material was undetectable. Post-reaction, acetonitrile was removed under reduced pressure, and the residue was extracted with ethyl acetate. The combined extracts were dried over anhydrous sodium sulfate and distilled under reduced pressure to yield a mixture. This mixture was dissolved in an LC-MS grade solvent, filtered through a 0.22 μm microporous membrane, and analyzed via LC-MS. An ion peak at 546.3443 m/z was observed in positive ion mode, suggesting a possible intermediate.Under the same conditions, BHT failed to capture the corresponding intermediate.

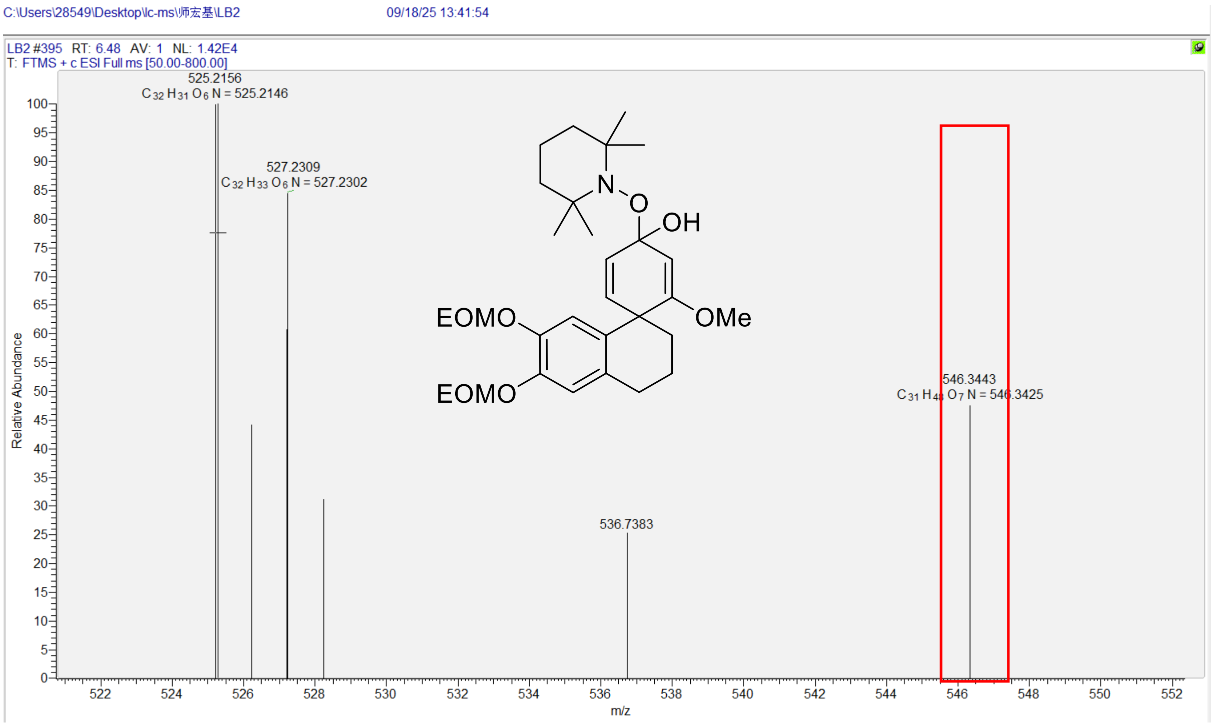

Figure 1. LC-MS analysis chart

Combined with control experiments and LC-MS analysis, the possible mechanism diagram was speculated.

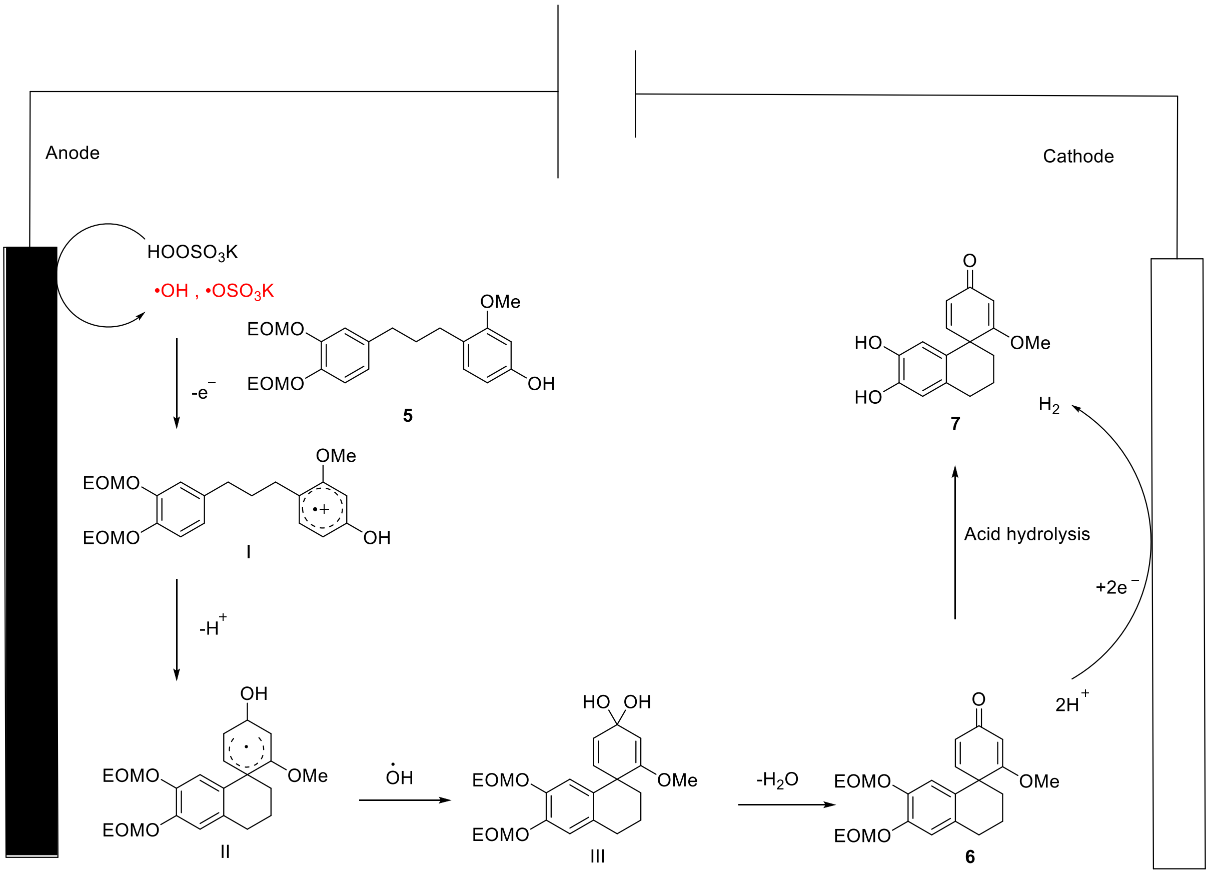

Figure 2. Proposed mechanism

III. Enantioseparation 7, 8 and Computational details

Chiral semi-preparative HPLC was performed on an Agilent 1100 HPLC with the CHIRALPAK® AD-H (4.6 mm × 250 mm) column. ECD calculation method: First, perform conformational search using Schrödinger, then conduct conformational analysis and ECD calculation using GaussView 6.0, and finally perform spectrum fitting using Origin 2021. Compound **7** is a racemate, and chiral separation was performed using an isopropanol/n - hexane (24% - 80%, 80min) system. LB-7A (t_R = 11.583); LB-7B (t_R = 18.477).

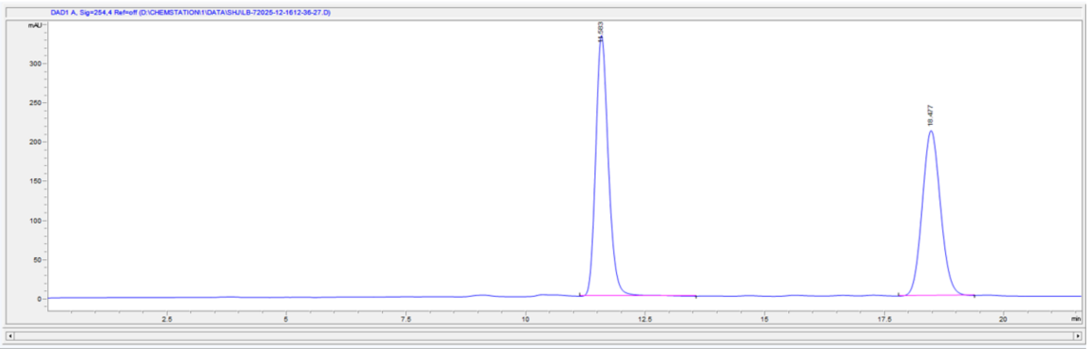

File information

LC-file	LB-72025-12-1612-36-27.D
File path	D:\CHEMSTATION\1\DATA\SHJ
Date	16-Dec-25, 12:38:00
Sample	LB-7
Sample information	
Barcode	
Operator	SYSTEM
Method	8 25% (Chiral splitting) .M
Reference	
Analysis time	21.613 min
Sampling frequency	0.0067 min (0.402 nec), 3243 Data point

#	RT	PA	k	W	PA%	S	Type
1	11.583	6165.6	333.3	0.2838	52.366	0.772	BB
2	18.477	5608.4	211.2	0.4119	47.634	0.9	BB

Figure 3. HPLC data of compound **7**

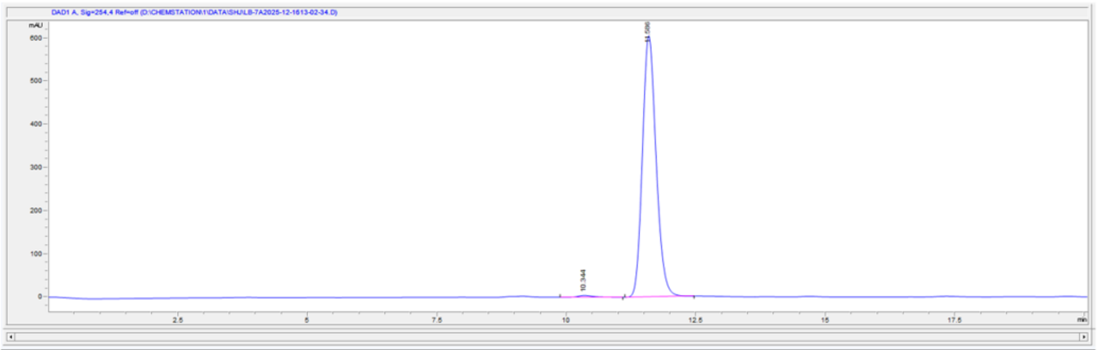

File information

LC-file	LB-7A2025-12-1613-02-34.D
File path	D:\CHEMSTATION\1\DATA\SHJ
Date	16-Oec-25, 13:04:04
Sample	LB-7A
Sample information	
Barcode	
Operator	SYSTEM
Method	B 25% (Chiral splitting) .M
Reference	
Analysis time	20.053 min
Sampling frequency	0.0067 min (0.402 sec), 3009 Data point

#	RT	PA	k	W	PA%	S	Type
1	10.344	82.9	4.1	0.3022	0.740	0.648	BB
2	11.586	11123.9	606.5	0.282	99.260	0.819	BB

Figure 4. HPLC data of compound **(+)-7**

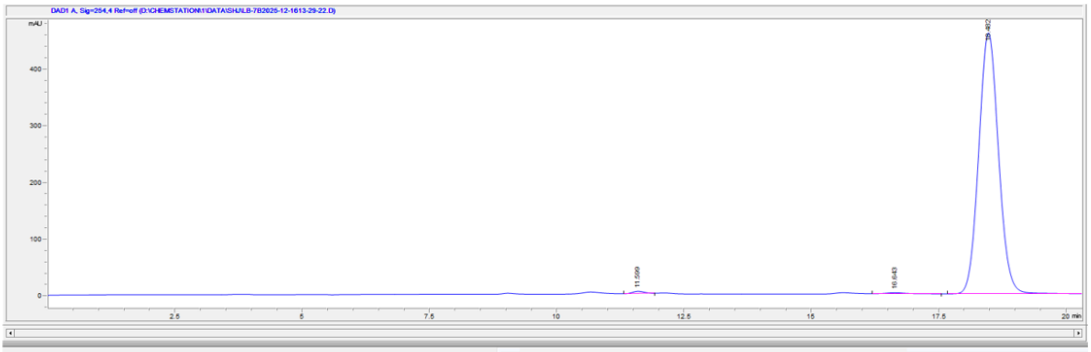

File information

LC-file	LB-7B2025-12-1613-29-22.D
File path	D:\CHEMSTATION\LIDATA\SHJ
Date	16-Dec-25, 13:30:55
Sample	LB-7B
Sample information	
Barcode	
Operator	SYSTEM
Method	B 25% (Chiral splitting) .M
Reference	
Analysis time	20.313 min
Sampling frequency	0.0067 min (0.402 sec), 3048 Data point

#	RT	PA	k	W	PA%	S	Type
1	11.599	61.4	3.8	0.2461	0.490	0.894	BB
2	16.643	45.2	1.7	0.3817	0.361	0.741	BB
3	18.482	12414.4	459.8	0.419	99.149	0.913	BBA

Figure 5. HPLC data of compound **(-)-7**

Rudolph Research Analytical

This sample was measured on an Autopol VI, Serial #91058
Manufactured by Rudolph Research Analytical, Hackettstown, NJ, USA.

Measurement Date : Sunday, 19-OCT-2025

Set Temperature : 25.0

Time Delay : Disabled

Delay between Measurement : Disabled

n	Average	Std.Dev.	% RSD	Maximum	Minimum
5	106.18	0.25	0.23	106.36	105.91

S.No	Sample ID	Time	Result	Scale	OR °Arc	WLG.nm	Lg.mm	Conc.g/100ml	Temp.
1	LB-7A	01:40:22 PM	106.36	SR	0.234	589	100.00	0.220	25.0
2	LB-7A	01:40:28 PM	106.36	SR	0.234	589	100.00	0.220	25.0
3	LB-7A	01:40:34 PM	105.91	SR	0.233	589	100.00	0.220	25.0
4	LB-7A	01:40:41 PM	105.91	SR	0.233	589	100.00	0.220	25.0
5	LB-7A	01:40:47 PM	106.36	SR	0.234	589	100.00	0.220	25.0

Rudolph Research Analytical

This sample was measured on an Autopol VI, Serial #91058
Manufactured by Rudolph Research Analytical, Hackettstown, NJ, USA.

Measurement Date : Sunday, 19-OCT-2025

Set Temperature : 25.0

Time Delay : Disabled

Delay between Measurement : Disabled

n	Average	Std.Dev.	% RSD	Maximum	Minimum
5	-136.00	0.00	0.00	-136.00	-136.00

S.No	Sample ID	Time	Result	Scale	OR °Arc	WLG.nm	Lg.mm	Conc.g/100ml	Temp
1	LB-7B	02:01:06 PM	-136.00	SR	-0.272	589	100.00	0.200	25.0
2	LB-7B	02:01:12 PM	-136.00	SR	-0.272	589	100.00	0.200	25.0
3	LB-7B	02:01:18 PM	-136.00	SR	-0.272	589	100.00	0.200	25.0
4	LB-7B	02:01:25 PM	-136.00	SR	-0.272	589	100.00	0.200	25.0
5	LB-7B	02:01:31 PM	-136.00	SR	-0.272	589	100.00	0.200	25.0

Figure 6. Optical rotation data of compound **7**

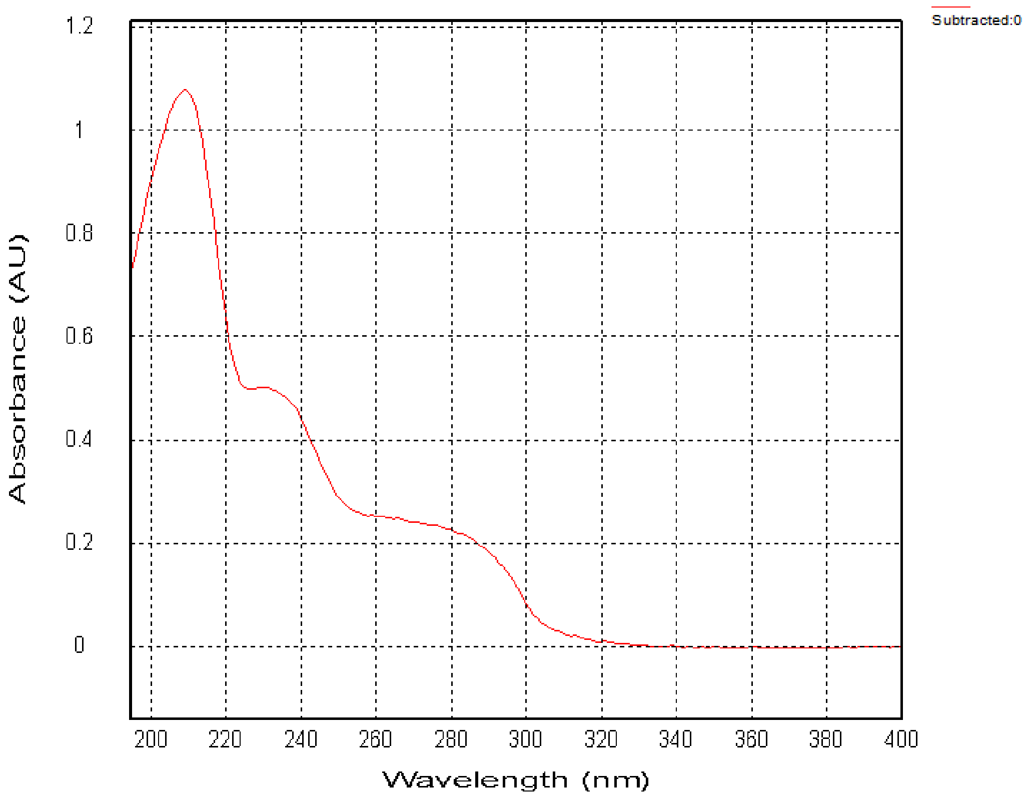

Figure 7. UV spectrum of compound **7**

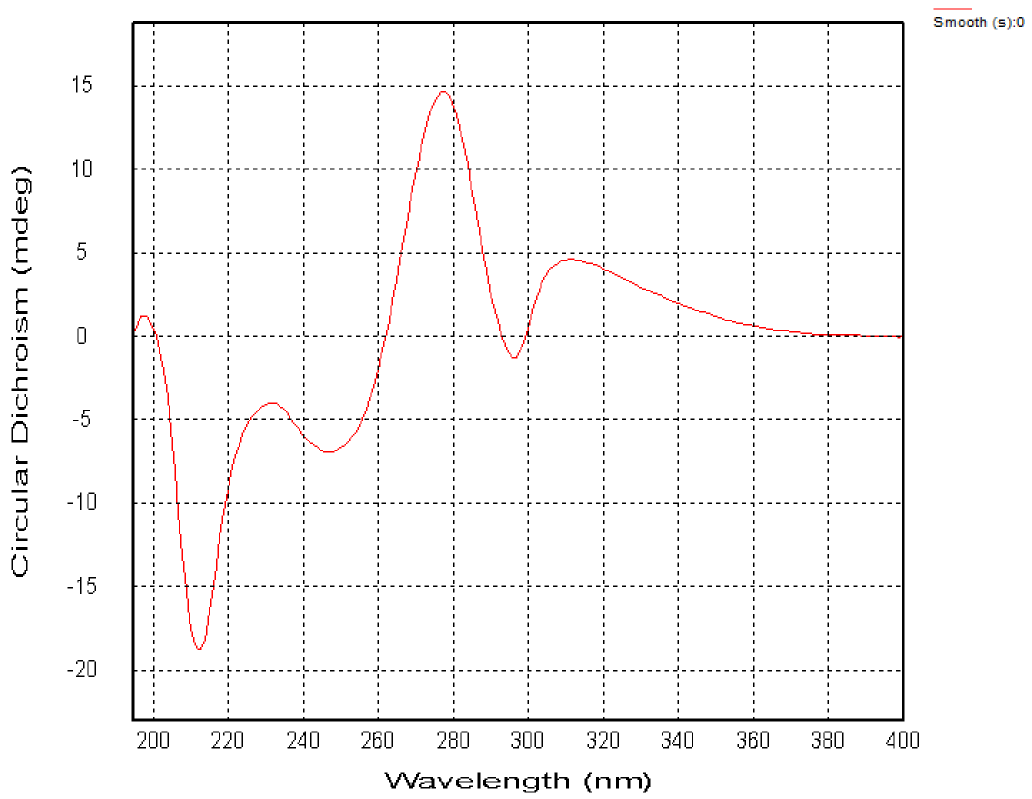

Figure 8. CD spectrum of (+)-**7**

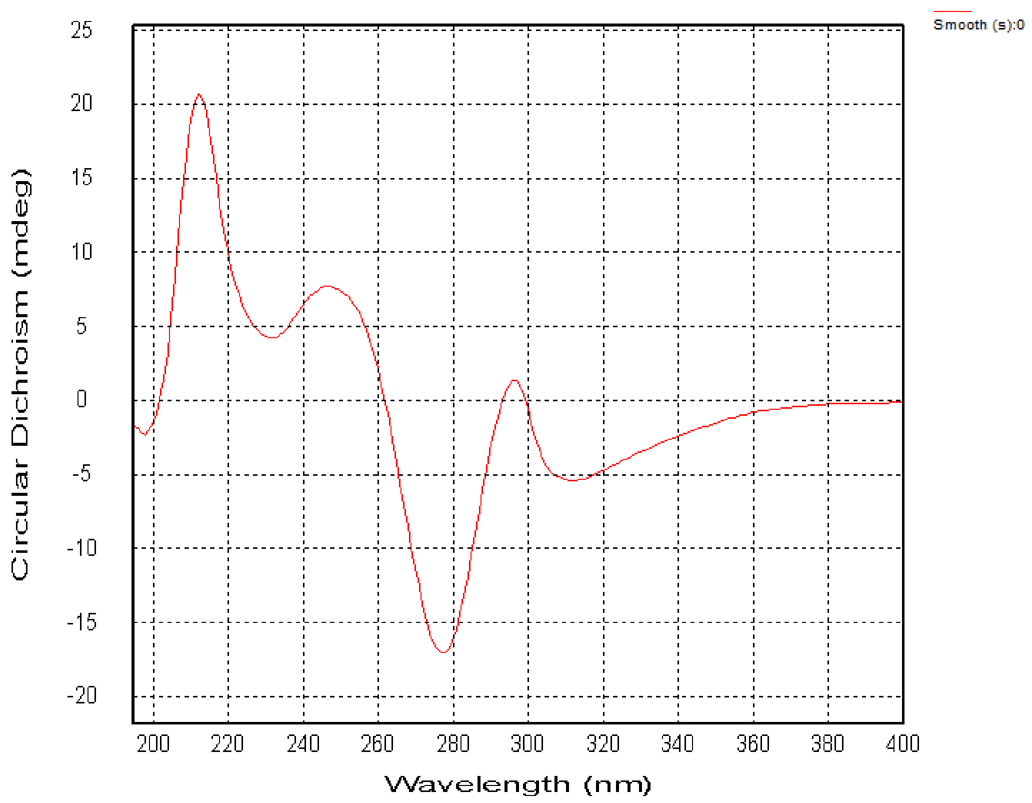

Figure 9. CD spectrum of (-)-**7**

Conformation 1 of compound **7**

0 1

C	-2.22294000	-1.52635800	-0.18033800
C	-3.26494900	-0.58771200	-0.08687800
C	-2.97480700	0.76639600	-0.03293400
C	-1.64895200	1.23187000	-0.05944600
C	-0.60846900	0.29496800	-0.14321000
C	-0.91241400	-1.07460900	-0.21624800
O	-2.49463400	-2.86184200	-0.24253000
O	-4.53432400	-1.12264300	-0.06574100
C	-1.39229300	2.72455400	0.02060300
C	-0.02143700	3.10764900	-0.54116500
C	0.87855200	0.71381100	-0.22463100
C	1.06159900	2.23531000	0.09539700
C	1.69664200	-0.07482700	0.78688400

C	2.75173200	-0.85595000	0.46156500
C	3.18962400	-1.03268200	-0.92798300
C	2.39673300	-0.32585000	-1.95892100
C	1.35657200	0.45390700	-1.63823100
O	4.15899300	-1.72913000	-1.22448000
O	1.23974300	0.12868400	2.04363900
C	1.88772400	-0.54412000	3.11913200
H	-3.79104900	1.48574000	0.02990900
H	-0.11938700	-1.81164100	-0.29912100
H	-3.46119200	-2.96395800	-0.21340300
H	-5.18677900	-0.40926000	-0.00191700
H	-1.44825800	3.05053600	1.07083300
H	-2.18845300	3.26534300	-0.50664600
H	-0.01819400	2.99586700	-1.63313100
H	0.18945000	4.16384600	-0.33445400
H	2.06204500	2.53857000	-0.23249900
H	1.02261100	2.36253900	1.18295400
H	3.32901200	-1.39313100	1.20509800
H	2.70742000	-0.47671600	-2.98902100
H	0.78798800	0.95557800	-2.41793600
H	1.82864700	-1.63137600	2.99295700
H	2.93939800	-0.24341300	3.19645500
H	1.35099900	-0.24723200	4.02124300

1 2 1.5 6 1.5 7 1.0

2 3 2.0 8 1.0

3 4 1.5 21 1.0

4 5 1.5 9 1.0

5 6 1.5 11 1.0

6 22 1.0

7 23 1.0

8 24 1.0

9 10 1.0 25 1.0 26 1.0

10 12 1.0 27 1.0 28 1.0

11 12 1.0 13 1.0 17 1.0

12 29 1.0 30 1.0

13 14 2.0 19 1.0

14 15 1.0 31 1.0

15 16 1.0 18 2.0

16 17 2.0 32 1.0

17 33 1.0

18

19 20 1.0

20 34 1.0 35 1.0 36 1.0

21

22

23

24

25

26

27

28

29

30

31

32

33

34

35

36

Conformation 2 of compound **7**

0 1

C	-2.21828200	-1.51635000	-0.12845300
C	-3.27460700	-0.59205100	-0.04743100
C	-2.98082000	0.76390400	-0.02968000
C	-1.65755600	1.23112000	-0.08200400
C	-0.60822300	0.30310900	-0.15069200
C	-0.90914000	-1.07004500	-0.18613700
O	-2.60105800	-2.84040800	-0.15029800
O	-4.56761000	-1.02043100	0.00303000
C	-1.40590200	2.72656900	-0.05137000
C	-0.04715200	3.09786900	-0.64930600
C	0.87670500	0.72476100	-0.25472000
C	1.05365000	2.25881400	0.00160000
C	1.69915800	-0.02368300	0.78377000
C	2.73092700	-0.84554300	0.48258700
C	3.14491500	-1.09937500	-0.90252300
C	2.36702500	-0.40872000	-1.95524600
C	1.34783800	0.40802400	-1.65860100
O	4.08273500	-1.84649300	-1.17741100
O	1.26666200	0.25037400	2.03520200
C	1.93021100	-0.36477100	3.13533800
H	-3.80787500	1.46694200	0.02088300
H	-0.09984800	-1.79471300	-0.25651700
H	-1.81916400	-3.40310200	-0.25502500
H	-4.55304000	-1.99210400	-0.03921200
H	-1.44597300	3.08288800	0.98943700
H	-2.21458600	3.24568900	-0.58016500
H	-0.06109100	2.94547500	-1.73641700
H	0.15967800	4.16240500	-0.48601100

H	2.04624400	2.55583500	-0.35507100
H	1.03212800	2.42884700	1.08350300
H	3.30721800	-1.35881600	1.24372700
H	2.66958500	-0.60348500	-2.98045300
H	0.78841300	0.89624100	-2.45336200
H	1.85492300	-1.45704700	3.07818200
H	2.98714700	-0.07521800	3.17051500
H	1.41792300	-0.00597100	4.02900500

1 2 1.5 6 2.0 7 1.0

2 3 1.5 8 1.0

3 4 1.5 21 1.0

4 5 1.5 9 1.0

5 6 1.5 11 1.0

6 22 1.0

7 23 1.0

8 24 1.0

9 10 1.0 25 1.0 26 1.0

10 12 1.0 27 1.0 28 1.0

11 12 1.0 13 1.0 17 1.0

12 29 1.0 30 1.0

13 14 2.0 19 1.0

14 15 1.0 31 1.0

15 16 1.0 18 2.0

16 17 2.0 32 1.0

17 33 1.0

18

19 20 1.0

20 34 1.0 35 1.0 36 1.0

21

22

23

24

25

26

27

28

29

30

31

32

33

34

35

36

Conformation 3 of compound **7**

0 1

C	-2.28242000	-1.53094400	0.12257300
C	-3.28119700	-0.54271900	0.08684200
C	-2.94271500	0.77621800	-0.17155000
C	-1.61047000	1.15748900	-0.40605200
C	-0.61424900	0.17099000	-0.37442300
C	-0.96531800	-1.16331200	-0.10818900
O	-2.60120000	-2.83206200	0.38112500
O	-4.56011900	-0.99431100	0.32835900
C	-1.30329400	2.61333800	-0.69940400
C	0.15225200	2.97336500	-0.39555700
C	0.87958500	0.48695200	-0.61479900
C	1.08668400	1.97233000	-1.07784700
C	1.66525600	0.23292300	0.66607600

C	2.73160600	-0.59585400	0.74441400
C	3.21505700	-1.35946800	-0.41114500
C	2.44169600	-1.20742900	-1.66420000
C	1.38660500	-0.38793900	-1.74209000
O	4.20047700	-2.09236600	-0.34562200
O	1.17145300	0.95156900	1.70088600
C	1.78560600	0.81241200	2.97921900
H	-3.72503400	1.53471200	-0.18957800
H	-0.20465100	-1.93746000	-0.07825600
H	-3.56425600	-2.87618400	0.50784200
H	-5.18103400	-0.25199100	0.28233200
H	-1.98458000	3.25576300	-0.12728600
H	-1.50980000	2.82484800	-1.76066100
H	0.37305300	3.98337700	-0.76270100
H	0.32244200	2.97268600	0.68467600
H	0.90717500	2.01531200	-2.16068600
H	2.13903700	2.23653700	-0.92791800
H	3.28523700	-0.74396700	1.66415300
H	2.77675400	-1.79860700	-2.51177100
H	0.82796400	-0.29310800	-2.67146300
H	1.73259800	-0.22578100	3.32648900
H	2.83347200	1.13404700	2.95138800
H	1.21864300	1.45706300	3.65226100

1 2 1.5 6 1.5 7 1.0

2 3 2.0 8 1.0

3 4 1.5 21 1.0

4 5 1.5 9 1.0

5 6 1.5 11 1.0

6 22 1.0

7 23 1.0

8 24 1.0

9 10 1.0 25 1.0 26 1.0

10 12 1.0 27 1.0 28 1.0

11 12 1.0 13 1.0 17 1.0

12 29 1.0 30 1.0

13 14 2.0 19 1.0

14 15 1.0 31 1.0

15 16 1.0 18 2.0

16 17 2.0 32 1.0

17 33 1.0

18

19 20 1.0

20 34 1.0 35 1.0 36 1.0

21

22

23

24

25

26

27

28

29

30

31

32

33

34

35

36

Conformation 4 of compound **7**

0 1

C	-2.27646400	-1.51029800	0.13316900
C	-3.29054200	-0.53687300	0.10325500
C	-2.94903900	0.78174200	-0.16110600
C	-1.61934200	1.16439100	-0.40184600
C	-0.61376500	0.18757700	-0.37361600
C	-0.96129200	-1.14818600	-0.10253900
O	-2.70451200	-2.79178100	0.40665100
O	-4.58869500	-0.88196400	0.33491600
C	-1.31845000	2.62050800	-0.70113100
C	0.13541200	2.99028200	-0.40107900
C	0.87811900	0.50781800	-0.62119000
C	1.07769700	1.99478700	-1.08121300
C	1.66927200	0.24897100	0.65536300
C	2.71003000	-0.61245300	0.73693500
C	3.16687600	-1.39828900	-0.41419500
C	2.40796700	-1.21618300	-1.67207400
C	1.37626400	-0.36702000	-1.75210200
O	4.11894700	-2.17383200	-0.34154200
O	1.20397300	0.99151600	1.68520600
C	1.83043800	0.86029900	2.95846700
H	-3.74275200	1.52381200	-0.17433700
H	-0.18380600	-1.90956200	-0.07776800
H	-1.94618300	-3.39500900	0.40527200
H	-4.60909300	-1.84037700	0.49969100
H	-2.00305600	3.26009100	-0.13084000
H	-1.52929600	2.82487900	-1.76269000
H	0.34950100	4.00038600	-0.77185600
H	0.30727300	2.99504400	0.67884100

H	0.90088200	2.03755400	-2.16440100
H	2.12807300	2.26553300	-0.92837100
H	3.26255100	-0.76932700	1.65597600
H	2.73317300	-1.81074400	-2.52125400
H	0.82627100	-0.25240400	-2.68442100
H	1.75357600	-0.16898900	3.32747800
H	2.88624400	1.15192300	2.91095700
H	1.28984600	1.53323600	3.62530400

1 2 1.5 6 2.0 7 1.0

2 3 1.5 8 1.0

3 4 1.5 21 1.0

4 5 1.5 9 1.0

5 6 1.5 11 1.0

6 22 1.0

7 23 1.0

8 24 1.0

9 10 1.0 25 1.0 26 1.0

10 12 1.0 27 1.0 28 1.0

11 12 1.0 13 1.0 17 1.0

12 29 1.0 30 1.0

13 14 2.0 19 1.0

14 15 1.0 31 1.0

15 16 1.0 18 2.0

16 17 2.0 32 1.0

17 33 1.0

18

19 20 1.0

20 34 1.0 35 1.0 36 1.0

21

Compound **8** is a racemate, and chiral separation was performed using an isopropanol/n - hexane (20% - 80%, 80min) system. LB-9A (t_R = 14.921); LB-9B (t_R = 22.343).

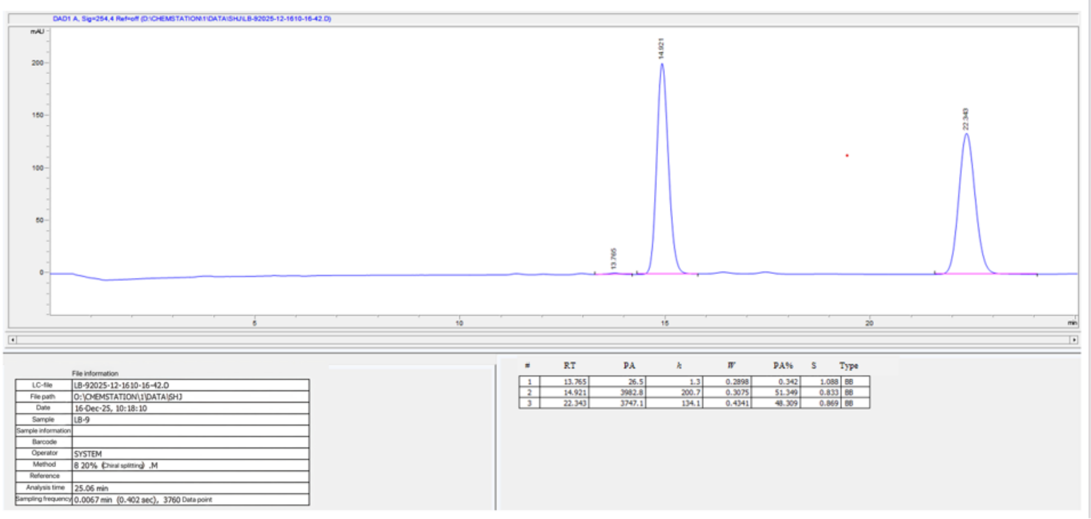

Figure 10. HPLC data of compound **8**

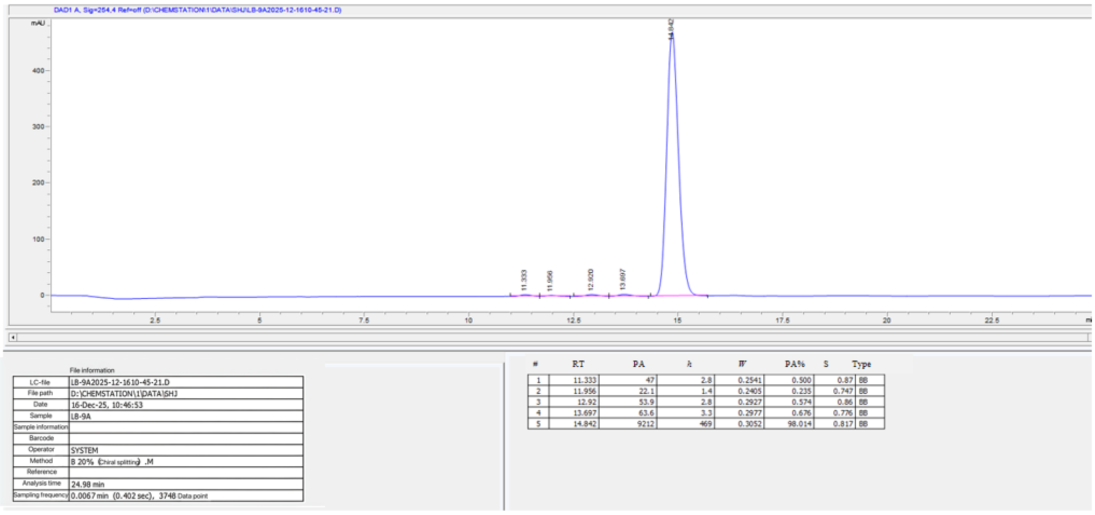

Figure 11. HPLC data of compound **(+)-8**

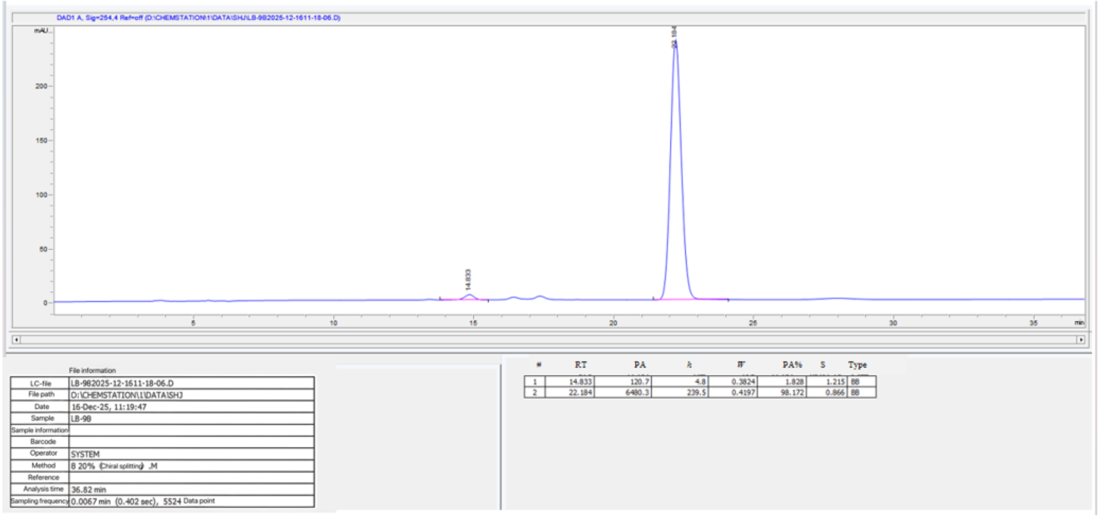

Figure 12. HPLC data of compound **(-)-8**

Rudolph Research Analytical

This sample was measured on an Autopol VI, Serial #91058
Manufactured by Rudolph Research Analytical, Hackettstown, NJ, USA.

Measurement Date : Sunday, 19-OCT-2025

Set Temperature : 25.0

Time Delay : Disabled

Delay between Measurement : Disabled

n	Average	Std.Dev.	% RSD	Maximum	Minimum
5	130.43	0.39	0.29	130.71	130.00

S.No	Sample ID	Time	Result	Scale	OR °Arc	WLG.nm	Lg.mm	Conc.g/100ml	Temp.
1	LB-9A	02:12:51 PM	130.71	SR	0.183	589	100.00	0.140	25.0
2	LB-9A	02:12:58 PM	130.71	SR	0.183	589	100.00	0.140	25.0
3	LB-9A	02:13:04 PM	130.00	SR	0.182	589	100.00	0.140	25.0
4	LB-9A	02:13:10 PM	130.71	SR	0.183	589	100.00	0.140	25.0
5	LB-9A	02:13:17 PM	130.00	SR	0.182	589	100.00	0.140	25.0

Rudolph Research Analytical

This sample was measured on an Autopol VI, Serial #91058
Manufactured by Rudolph Research Analytical, Hackettstown, NJ, USA.

Measurement Date : Sunday, 19-OCT-2025

Set Temperature : 25.0

Time Delay : Disabled

Delay between Measurement : Disabled

n	Average	Std.Dev.	% RSD	Maximum	Minimum
5	-98.11	0.30	-0.30	-97.78	-98.33

S.No	Sample ID	Time	Result	Scale	OR °Arc	WLG.nm	Lg.mm	Conc.g/100ml	Temp.
1	LB-9B	02:24:40 PM	-97.78	SR	-0.176	589	100.00	0.180	25.0
2	LB-9B	02:24:47 PM	-98.33	SR	-0.177	589	100.00	0.180	25.0
3	LB-9B	02:24:53 PM	-98.33	SR	-0.177	589	100.00	0.180	25.0
4	LB-9B	02:24:59 PM	-97.78	SR	-0.176	589	100.00	0.180	25.0
5	LB-9B	02:25:06 PM	-98.33	SR	-0.177	589	100.00	0.180	25.0

Figure 13. Optical rotation data of compound **8**

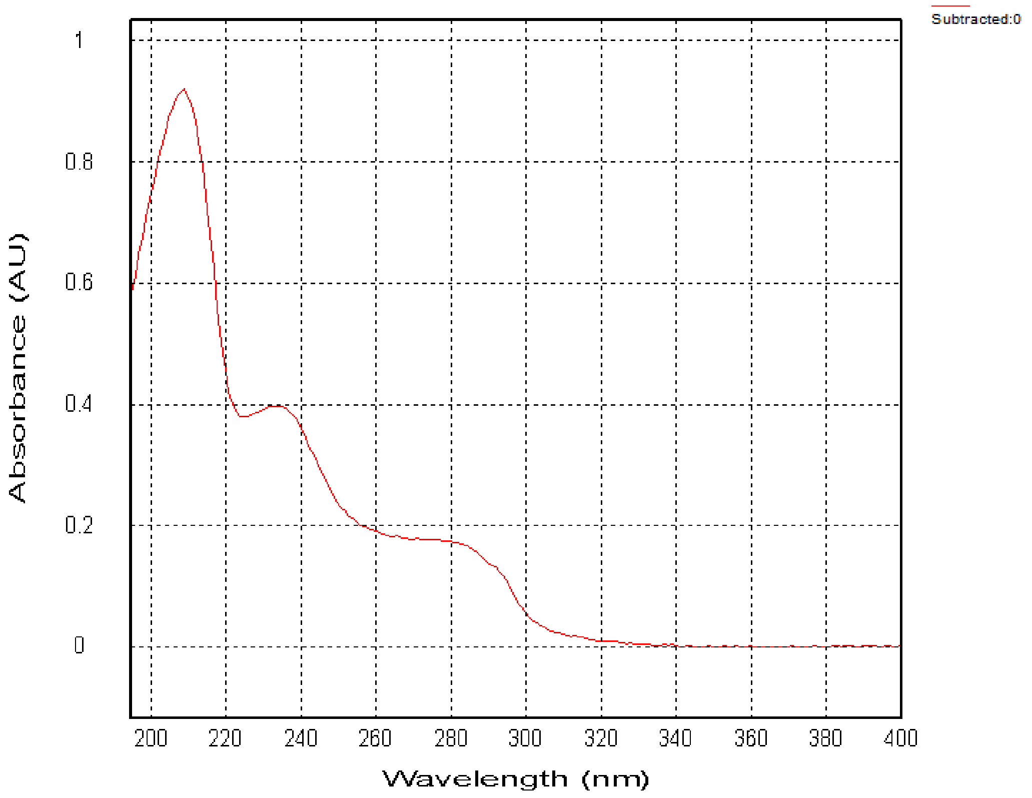

Figure 14. UV spectrum of compound **8**

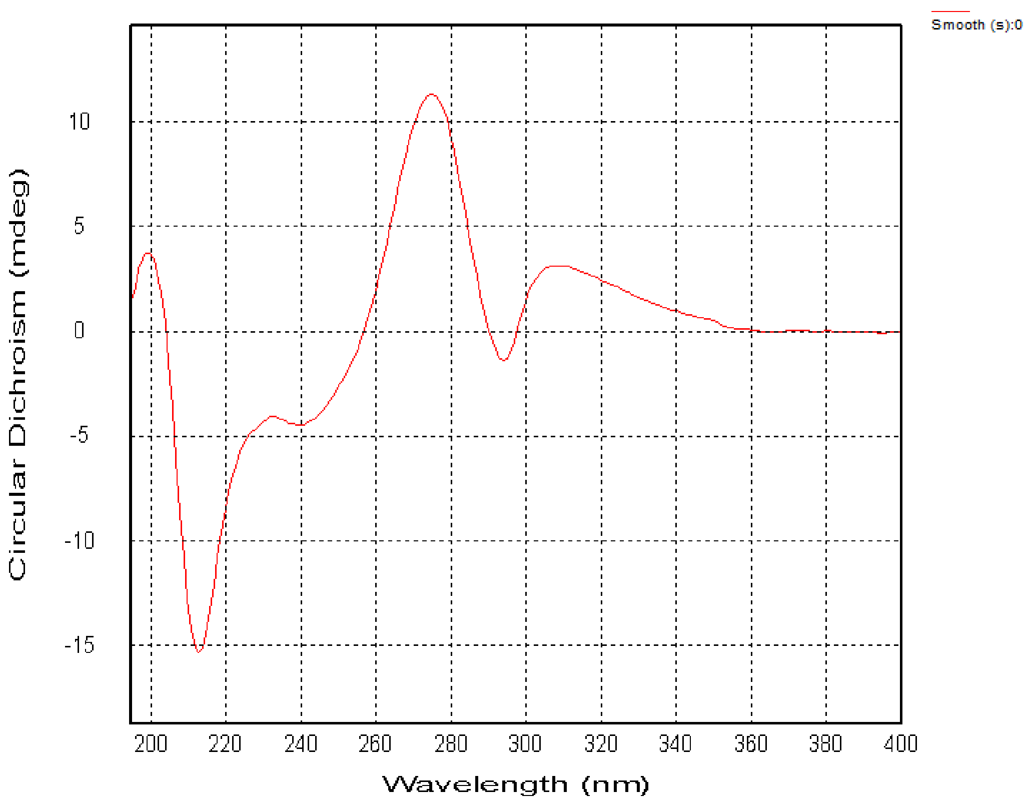

Figure 15. CD spectrum of (+)-**8**

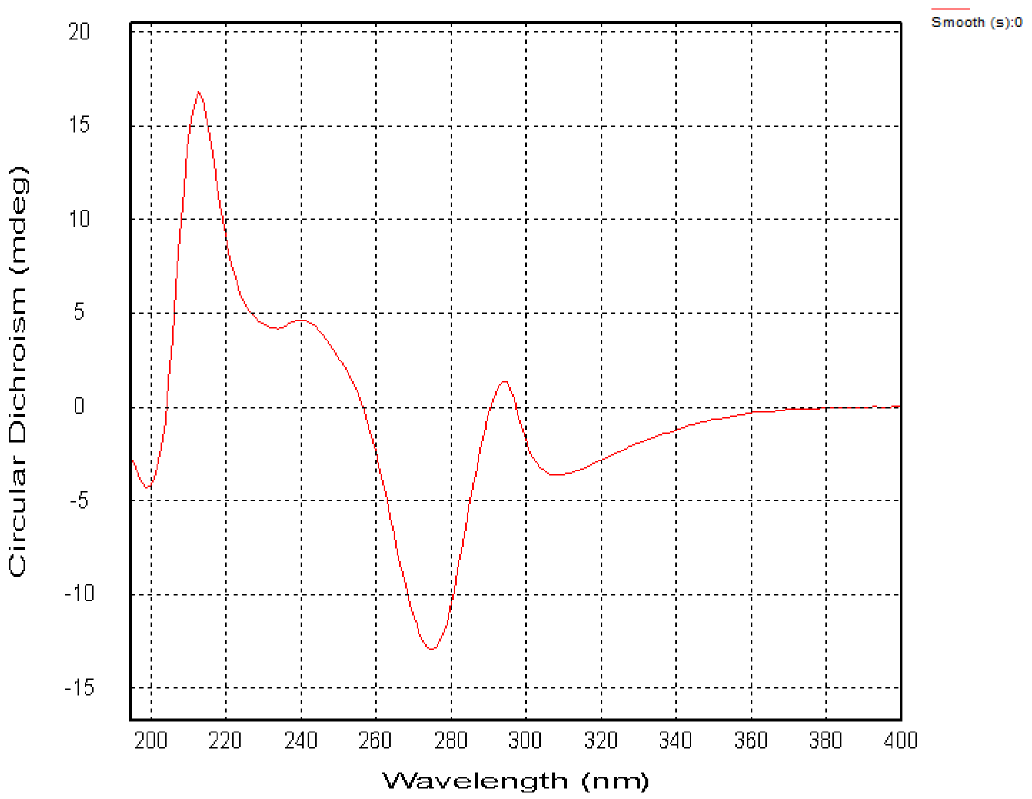

Figure 16. CD spectrum of (-)-**8**

Conformation 1 of compound **8**

0 1

C	-1.32515700	1.26010300	-0.06261900
C	-0.29788400	0.31162700	-0.14565400
C	1.19491600	0.70968500	-0.22330200
C	1.39849500	2.22783300	0.09982100
C	0.32872600	3.11569400	-0.53763500
C	-1.04862100	2.74948300	0.01941400
C	-2.66178600	0.81816100	-0.03785500
C	-2.97141300	-0.53280500	-0.09378300
C	-1.93592400	-1.48521800	-0.18735000
C	-0.62021000	-1.05469900	-0.22089700
C	1.99987300	-0.09217600	0.78825700
C	3.04466100	-0.88740400	0.46367100
C	3.48272200	-1.06794700	-0.92524900
C	2.70182700	-0.34833700	-1.95643200
C	1.67210400	0.44543100	-1.63632700
O	4.44289000	-1.77744800	-1.22109500
O	-2.22965400	-2.81513300	-0.25149100
O	-4.23126700	-1.08255100	-0.07710200
C	-5.34394900	-0.20628600	0.01220900
O	1.54341900	0.11527700	2.04457200
C	2.17920800	-0.56927100	3.11977600
H	1.35872100	2.35362000	1.18752500
H	2.40380300	2.51796200	-0.22522200
H	0.33357000	3.00594700	-1.62983200
H	0.55321400	4.16863900	-0.32838200
H	-1.83575600	3.30098800	-0.51036100
H	-1.10416700	3.07610300	1.06957600
H	-3.45383900	1.55805000	0.02488000

H	0.16220100	-1.80296600	-0.30348000
H	3.61287900	-1.43391100	1.20739600
H	3.01243600	-0.50175700	-2.98618900
H	1.11214800	0.95638500	-2.41623700
H	-3.19924100	-2.89184500	-0.22239300
H	-6.23125600	-0.84182500	0.01449000
H	-5.31340600	0.38205900	0.93826300
H	-5.38449500	0.47420900	-0.84799500
H	1.64465400	-0.26703600	4.02143000
H	2.10501400	-1.65527800	2.99077700
H	3.23487500	-0.28374400	3.20039900

1 2 1.5 6 1.0 7 1.5

2 3 1.0 10 1.5

3 4 1.0 11 1.0 15 1.0

4 5 1.0 22 1.0 23 1.0

5 6 1.0 24 1.0 25 1.0

6 26 1.0 27 1.0

7 8 1.5 28 1.0

8 9 1.5 18 1.0

9 10 2.0 17 1.0

10 29 1.0

11 12 2.0 20 1.0

12 13 1.0 30 1.0

13 14 1.0 16 2.0

14 15 2.0 31 1.0

15 32 1.0

16

17 33 1.0

18 19 1.0

19 34 1.0 35 1.0 36 1.0

20 21 1.0

21 37 1.0 38 1.0 39 1.0

22

23

24

25

26

27

28

29

30

31

32

33

34

35

36

37

38

39

Conformation 2 of compound **8**

0 1

C	-1.29613400	1.16082200	-0.44181500
C	-0.30278400	0.17495100	-0.39385700
C	1.19515200	0.48708300	-0.61209900
C	1.41326200	1.97247100	-1.06990200
C	0.46956800	2.97423800	-0.40170900
C	-0.98139600	2.61658800	-0.72922300
C	-2.63716100	0.78767800	-0.22986600

C	-2.98346700	-0.53142000	0.02386800
C	-1.98090000	-1.52140300	0.07644600
C	-0.66051300	-1.15959300	-0.13226200
C	1.96183100	0.22866300	0.67925900
C	3.02417500	-0.60387600	0.77161200
C	3.52137000	-1.36760100	-0.37797900
C	2.76654900	-1.21104500	-1.64165800
C	1.71543800	-0.38782700	-1.73327400
O	4.50329100	-2.10404200	-0.29942200
O	-2.31006500	-2.81991400	0.33087000
O	-4.25135200	-1.01328000	0.24751300
C	-5.33327500	-0.09557200	0.21304500
O	1.45618500	0.94777200	1.70801200
C	2.05190300	0.80479100	2.99448200
H	2.46361700	2.23429000	-0.90254700
H	1.25147100	2.01715800	-2.15546000
H	0.69811900	3.98436900	-0.76391900
H	0.62238000	2.97185600	0.68116400
H	-1.17078400	2.83024400	-1.79334800
H	-1.67045300	3.25963200	-0.16712300
H	-3.40305000	1.55637500	-0.26457400
H	0.09744100	-1.93573900	-0.08930400
H	3.56432200	-0.75494800	1.69886400
H	3.11165600	-1.80211400	-2.48527900
H	1.17036500	-0.28977700	-2.67034200
H	-3.27647400	-2.84994900	0.44013600
H	-6.23365000	-0.68059500	0.40875100
H	-5.22459600	0.67680600	0.98523200
H	-5.41611100	0.38478400	-0.77034100
H	1.47781800	1.45047400	3.66048900

H	1.99057600	-0.23368900	3.33958100
H	3.10117700	1.12284800	2.98181800

1 2 1.5 6 1.0 7 1.5

2 3 1.0 10 1.5

3 4 1.0 11 1.0 15 1.0

4 5 1.0 22 1.0 23 1.0

5 6 1.0 24 1.0 25 1.0

6 26 1.0 27 1.0

7 8 1.5 28 1.0

8 9 1.5 18 1.0

9 10 2.0 17 1.0

10 29 1.0

11 12 2.0 20 1.0

12 13 1.0 30 1.0

13 14 1.0 16 2.0

14 15 2.0 31 1.0

15 32 1.0

16

17 33 1.0

18 19 1.0

19 34 1.0 35 1.0 36 1.0

20 21 1.0

21 37 1.0 38 1.0 39 1.0

22

23

24

25

26

27

VI. Copies of ^1H, ^{13}C NMR Spectra, IR and HRMS

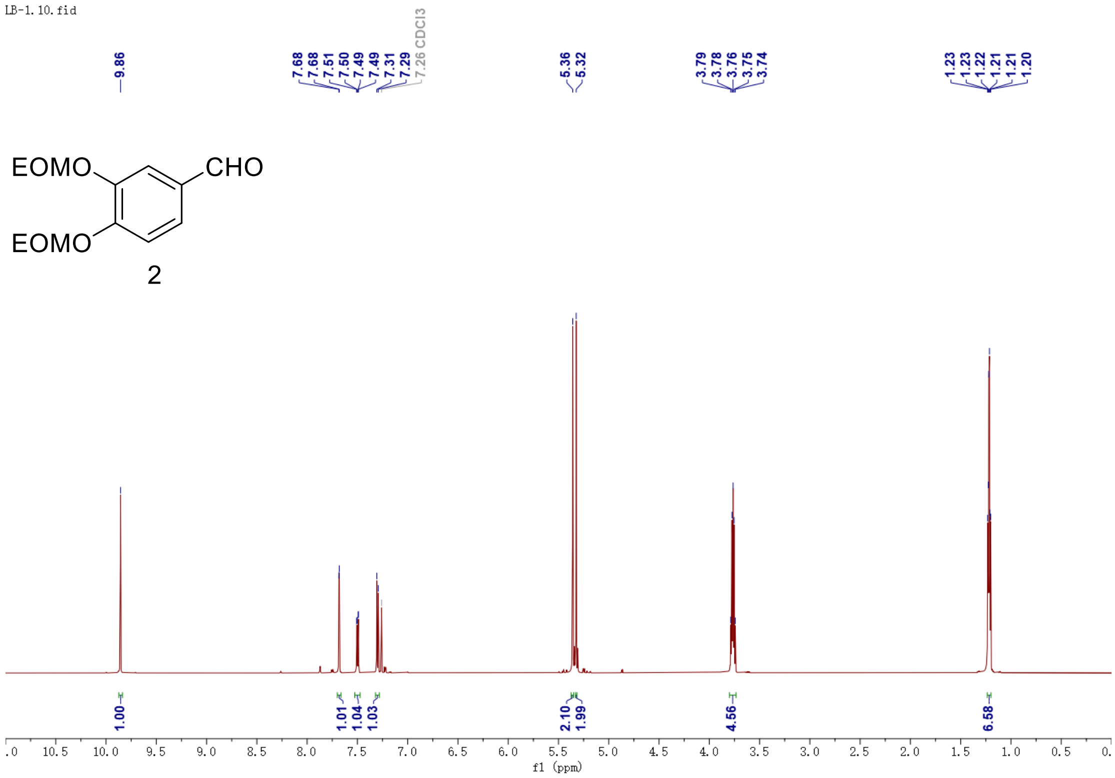

Figure 17. ^1H NMR (600 MHz, $CDCl_3$) spectrum of compound **2**

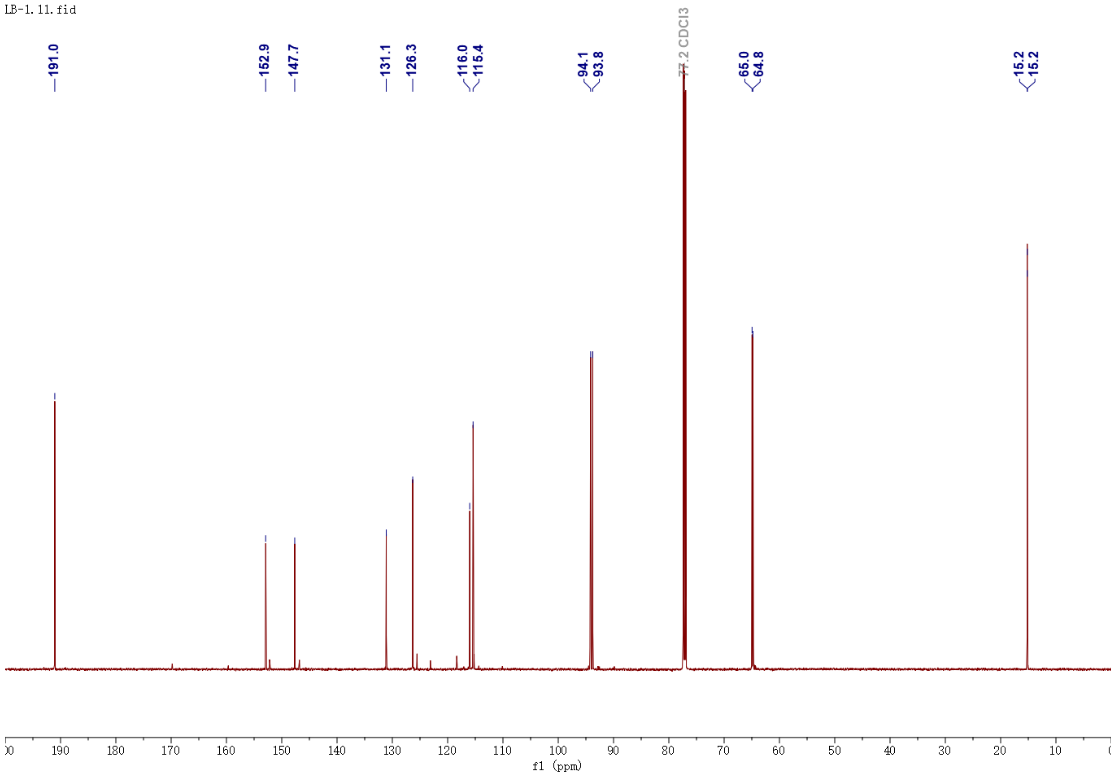

Figure 18. ^{13}C NMR (150 MHz, $CDCl_3$) spectrum of compound **2**

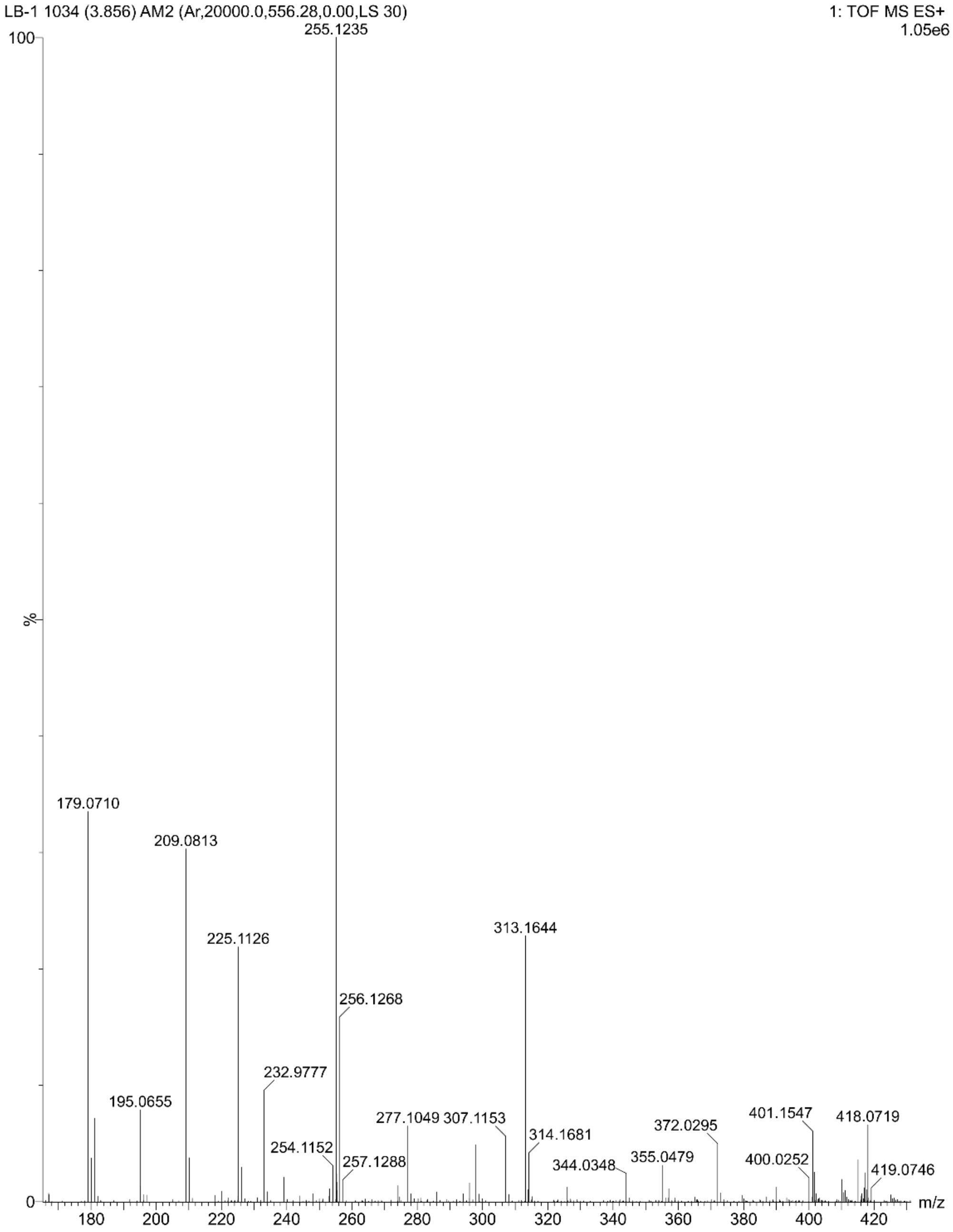

Figure 19. HRMS spectrum of compound **2**

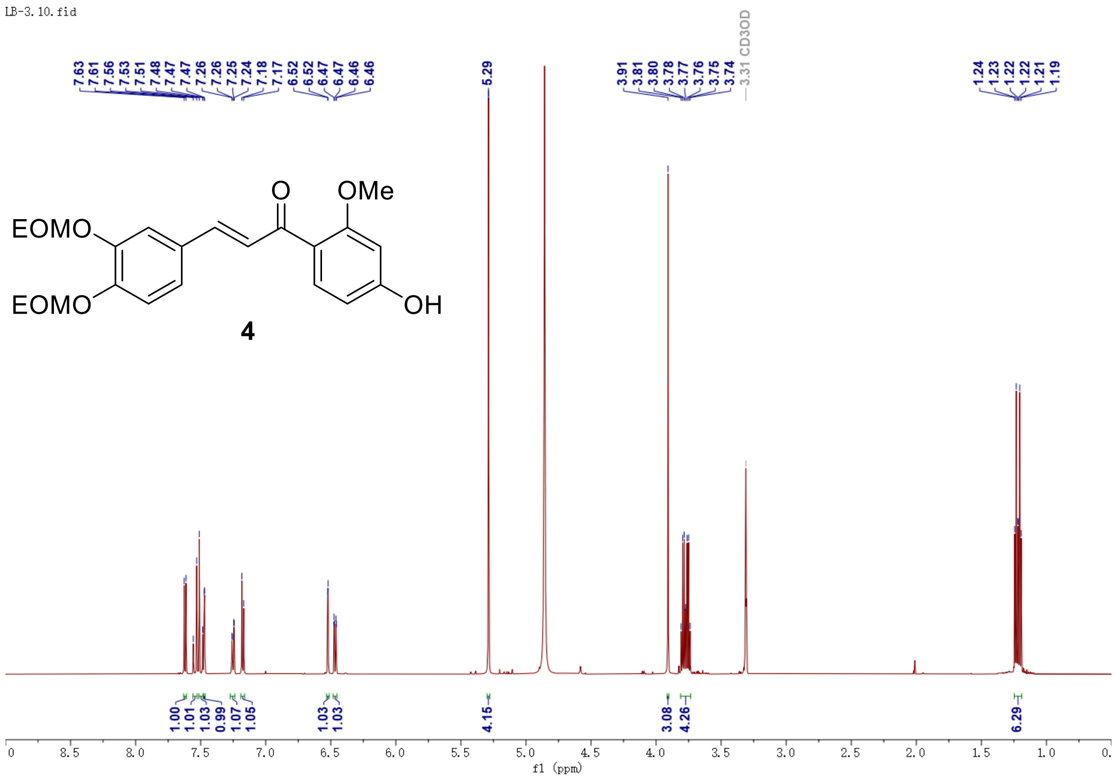

Figure 20. ^{1}H NMR (600 MHz, CD_3OD) spectrum of compound **4**

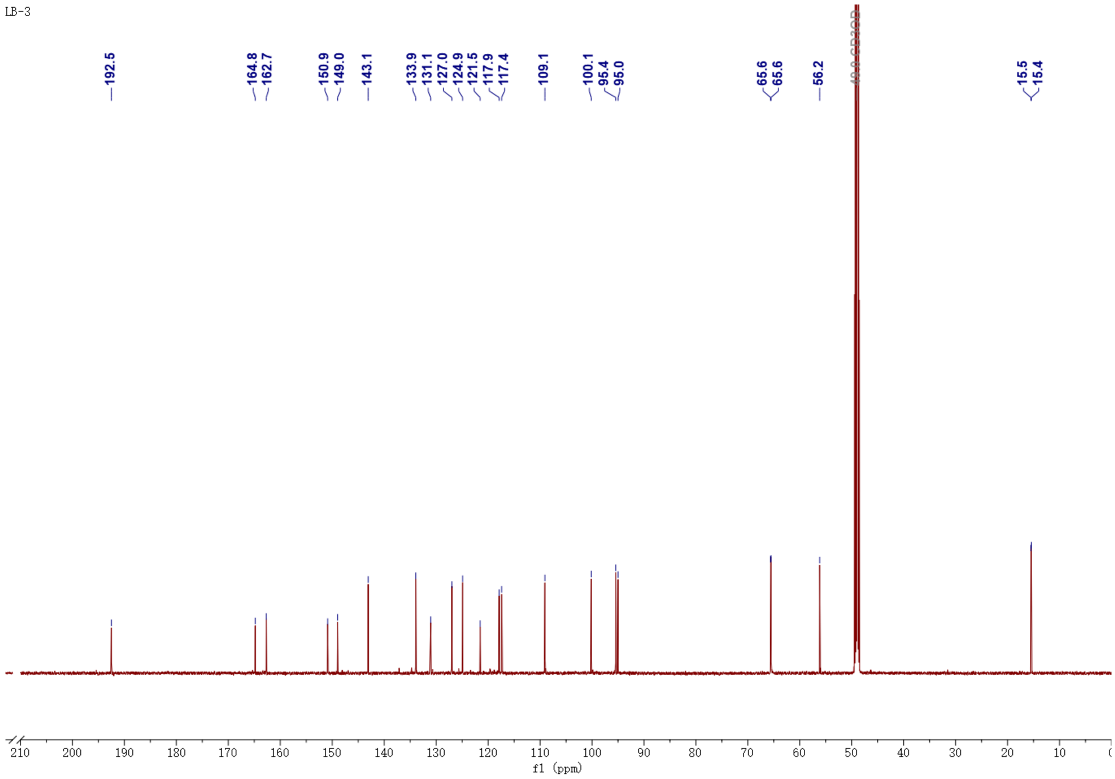

Figure 21. ^{13}C NMR (150 MHz, CD_3OD) spectrum of compound **4**

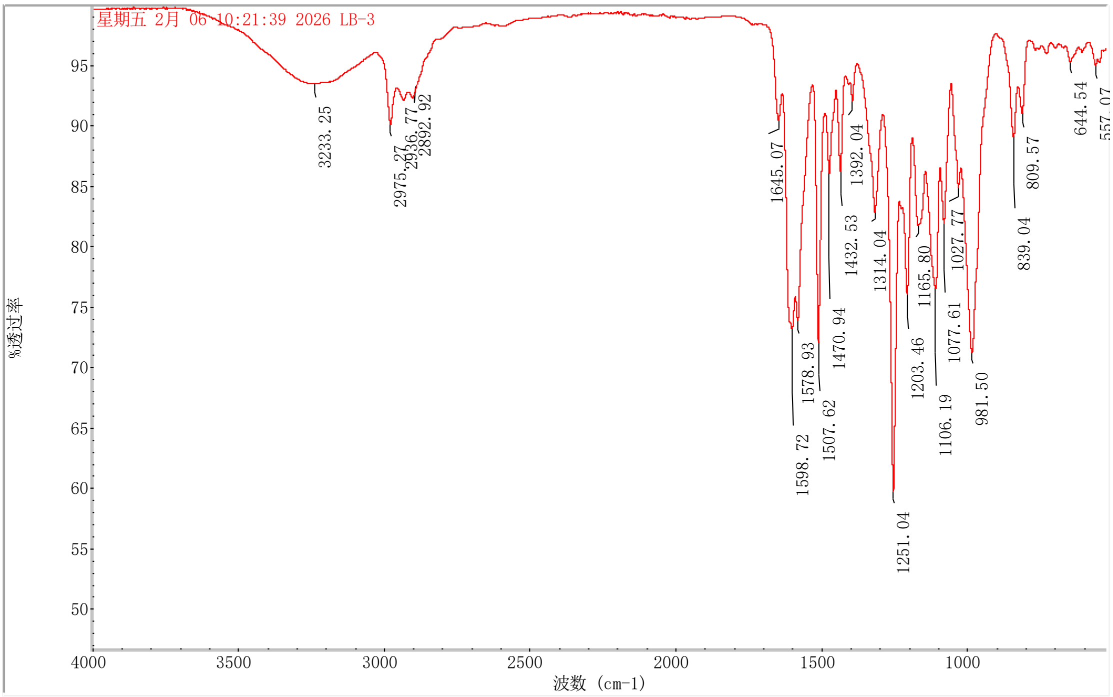

Figure 22. IR spectrum of compound **4**

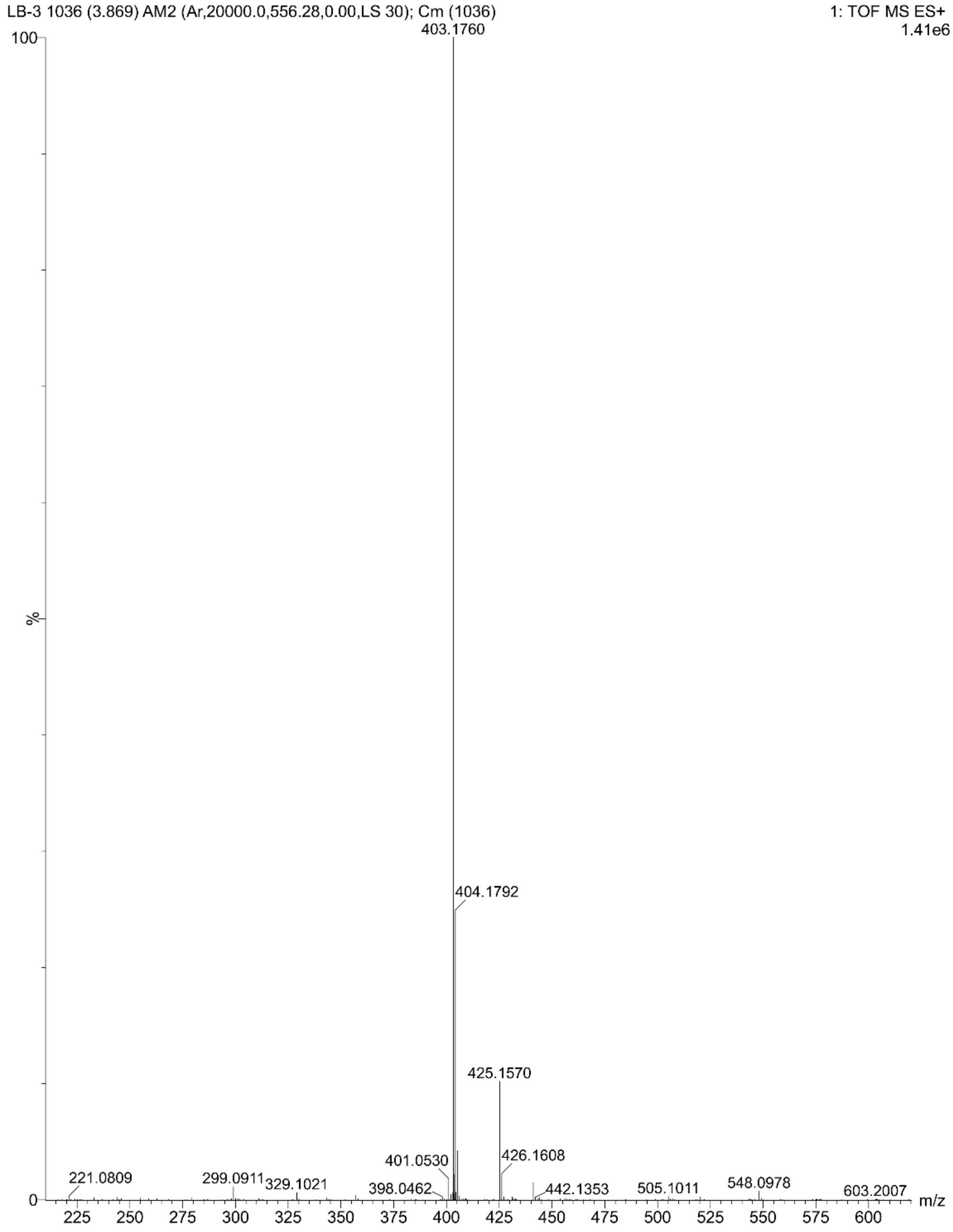

Figure 23. HRMS spectrum of compound **4**

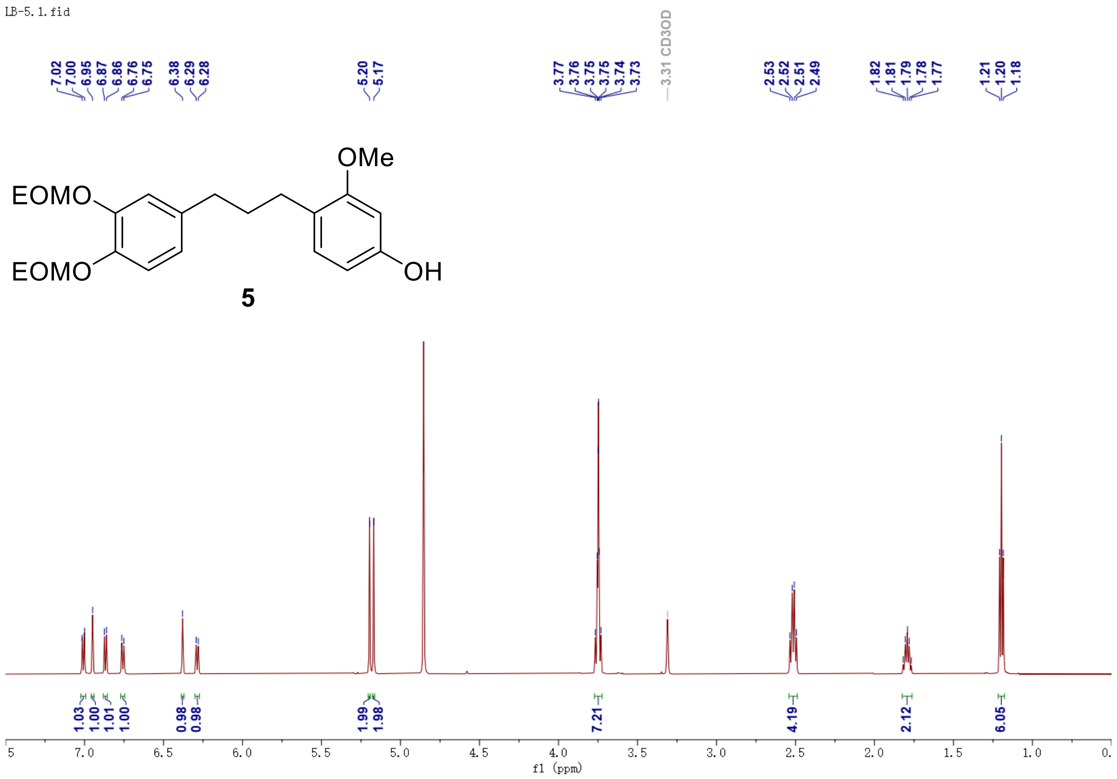

Figure 24. ^{1}H NMR (600 MHz, CD_3OD) spectrum of compound **5**

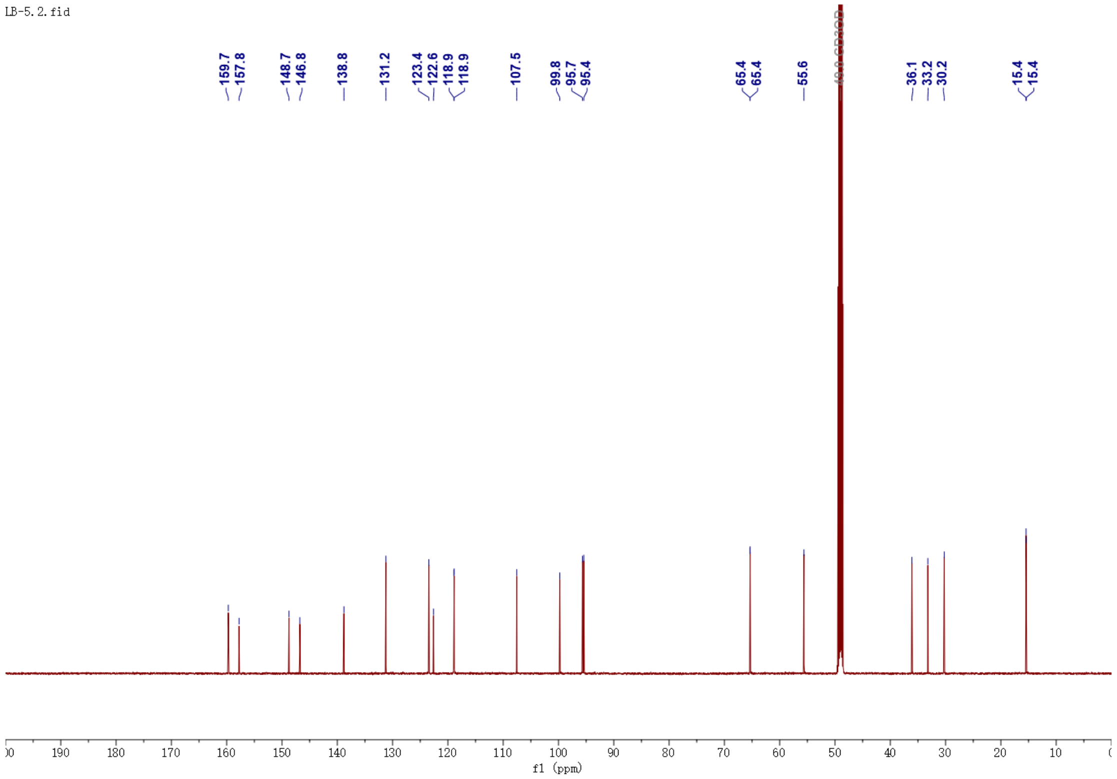

Figure 25. ^{13}C NMR (150 MHz, CD_3OD) spectrum of compound **5**

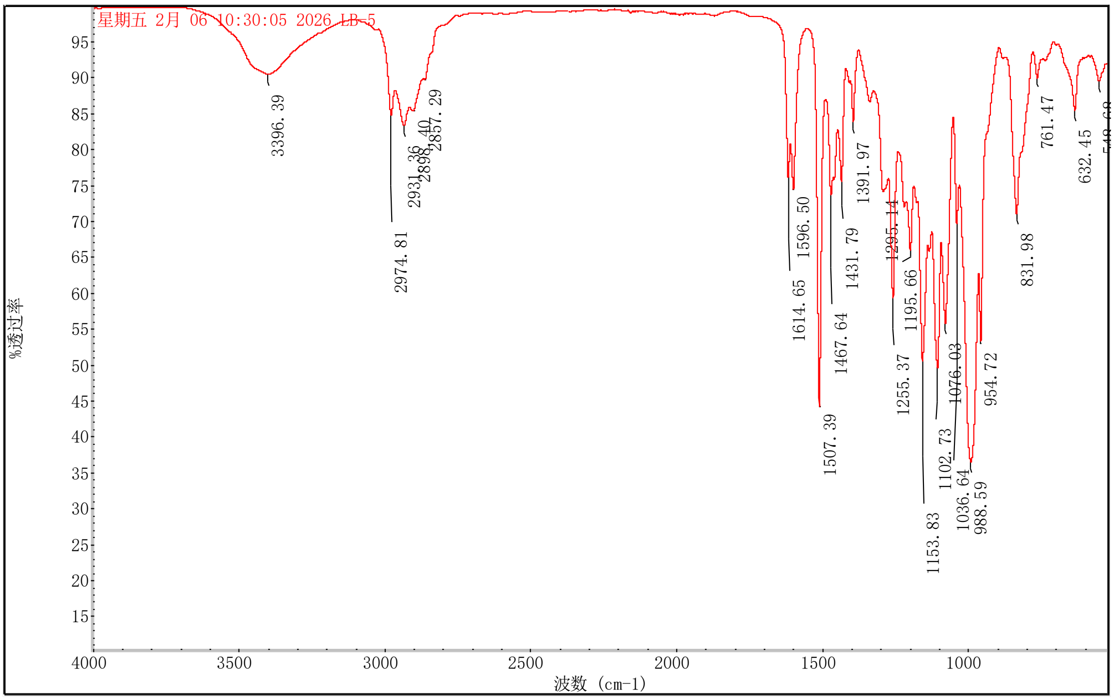

Figure 26. IR spectrum of compound **5**

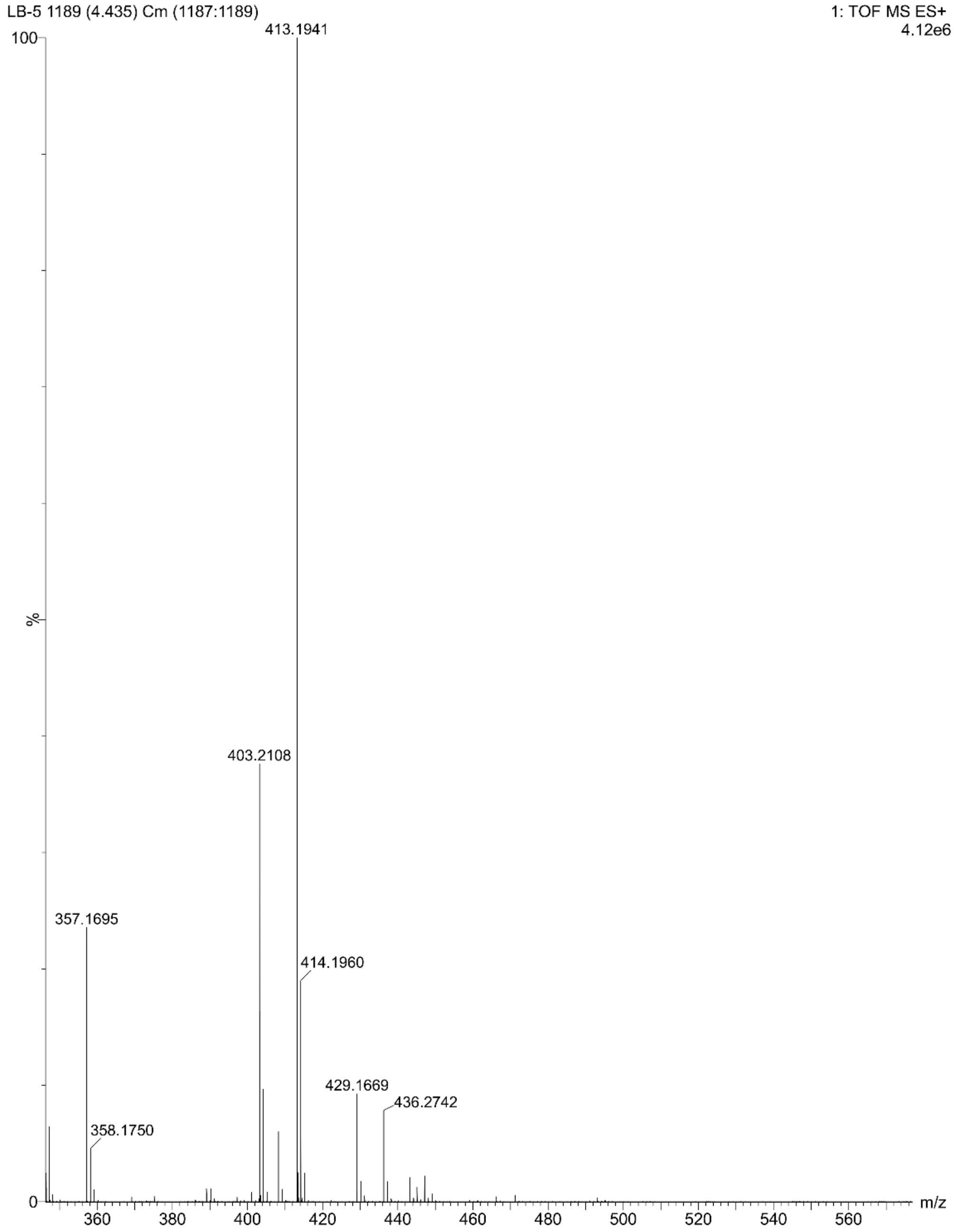

Figure 27. HRMS spectrum of compound **5**

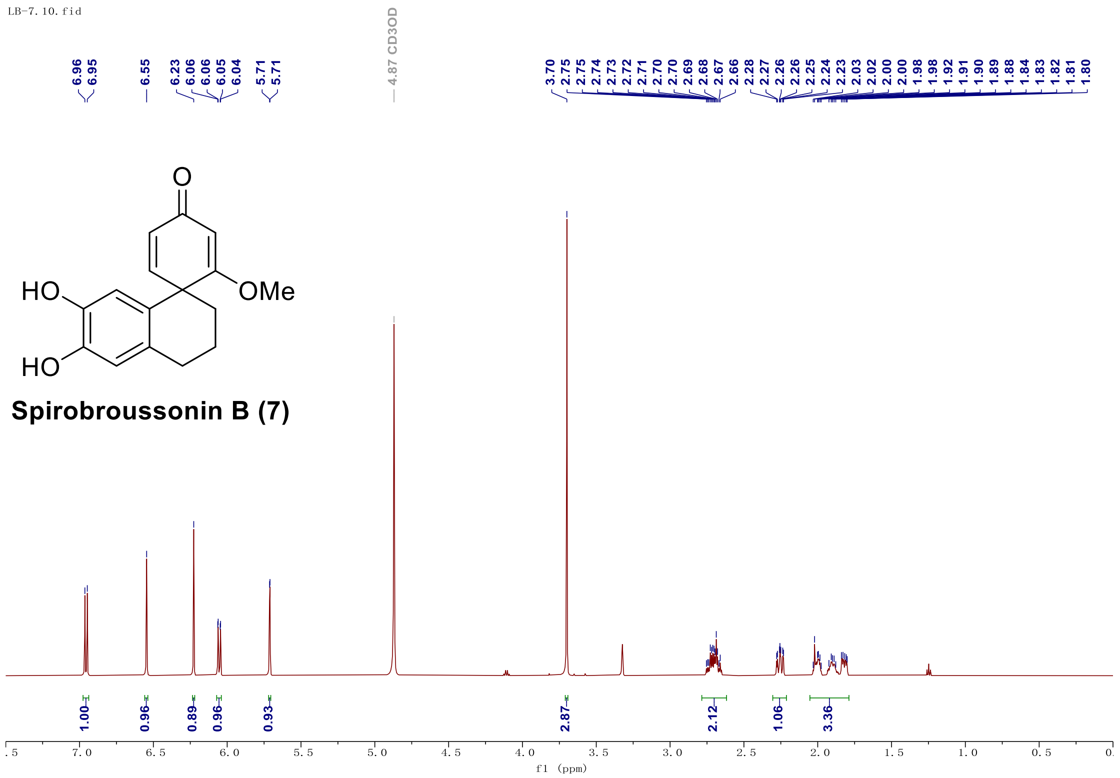

Figure 28. ^{1}H NMR (600 MHz, CD_3OD) spectrum of compound **7**

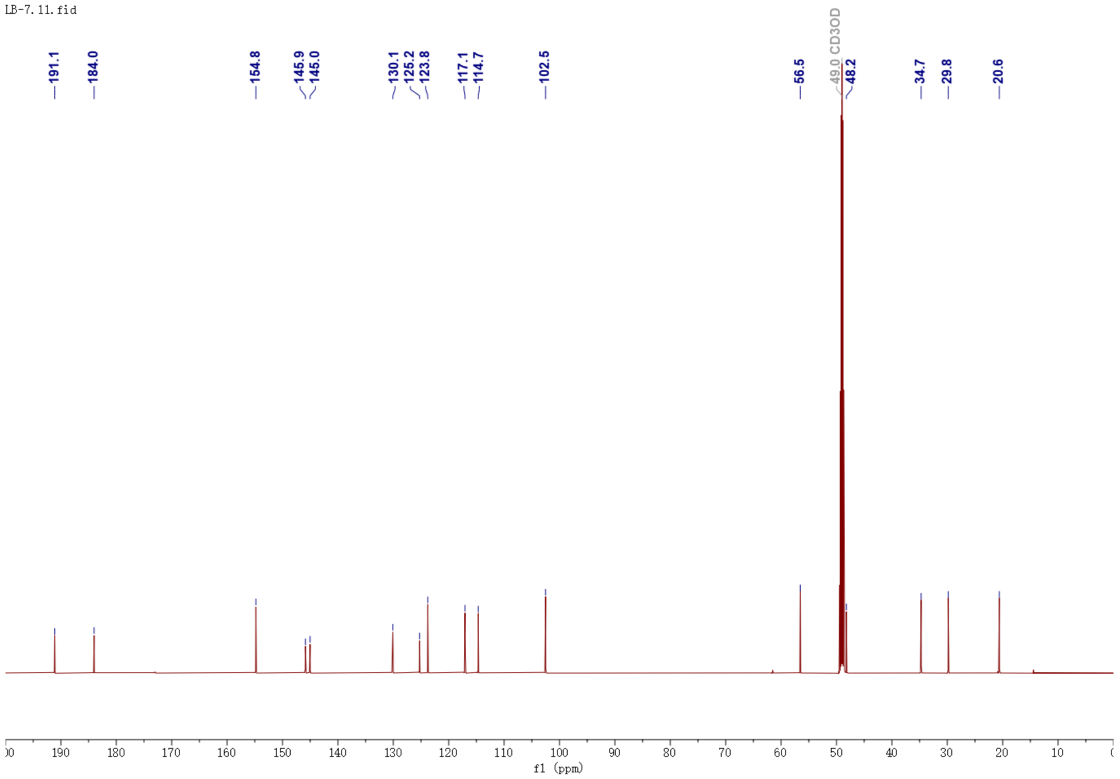

Figure 29. ^{13}C NMR (150 MHz, CD_3OD) spectrum of compound **7**

Table 1 Comparison of NMR data of natural and synthetic **7**

NO.	^{1}H NMR δ (J /Hz)		^{13}C NMR δ	
	Natural (DMSO-d_6)	Synthesis (MeOH-d_4)	Natural (DMSO-d_6)	Synthesis (MeOH-d_4)
1	-	-	143.7	145.0
2	-	-	144.5	145.9
3	6.17 (s)	6.21 (s)	113.6	114.7
4	-	-	122.9	123.8
5	-	-	127.7	130.1
6	6.48 (s)	6.53 (s)	116.1	117.1
7	-	-	45.9	48.2
8	1.8-2.0 (m)	1.8-2.25 (m)	28.2	29.8
9			19.0	20.6
10	2.62 (m)	2.69 (m)	33.1	34.7
11	5.94 (dd, 10.0, 1.5)	6.04 (dd, 9.8, 1.6)	151.8	154.8
12	6.89 (d, 10.0)	6.94 (d, 9.8)	123.9	125.2
13	-	-	186.9	191.1
14	5.65 (d, 1.5)	5.70 (d, 1.6)	101.9	102.5
15	-	-	179.9	184.0
16	3.63 (s)	3.69 (s)	55.8	56.5

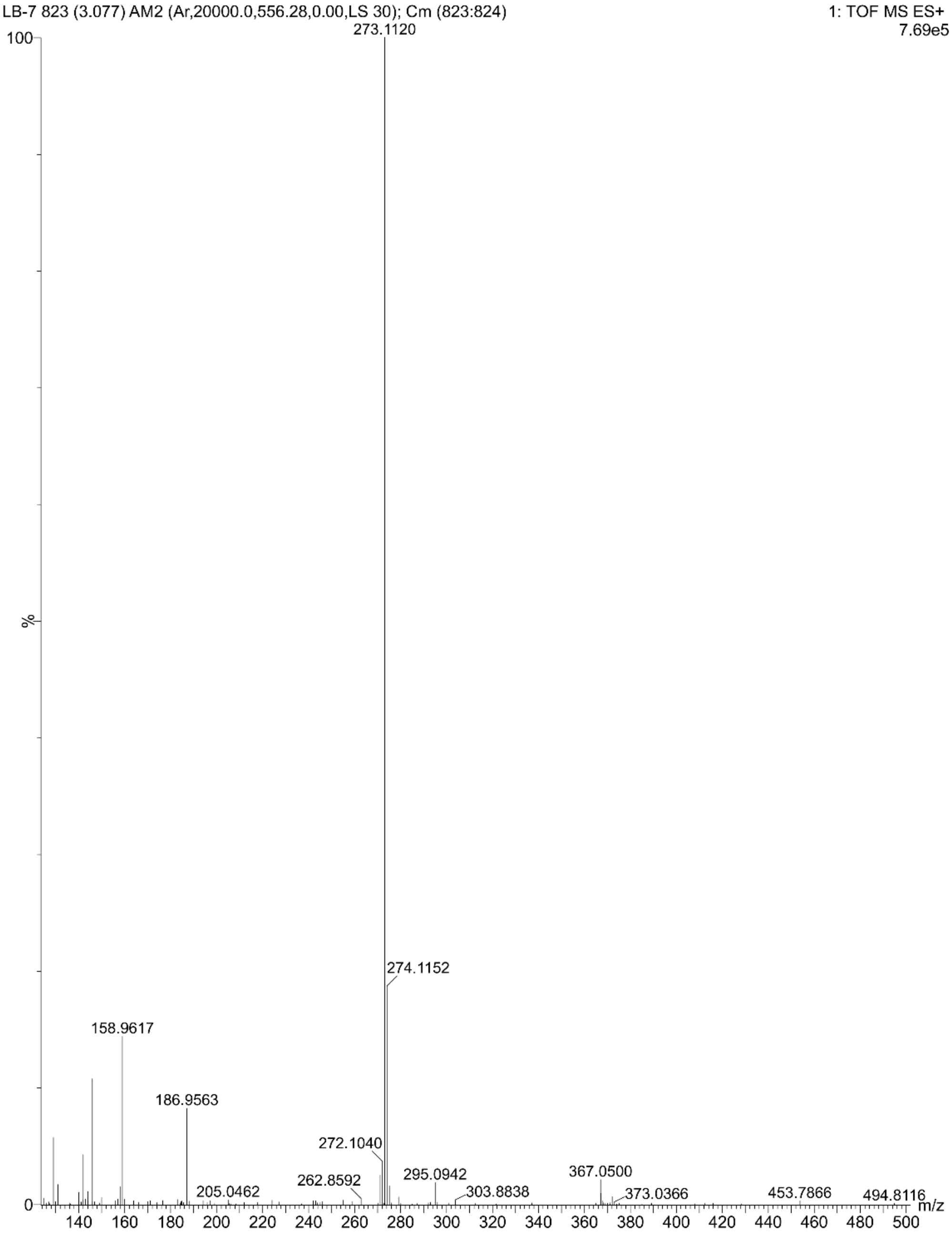

Figure 30. HRMS spectrum of compound **7**

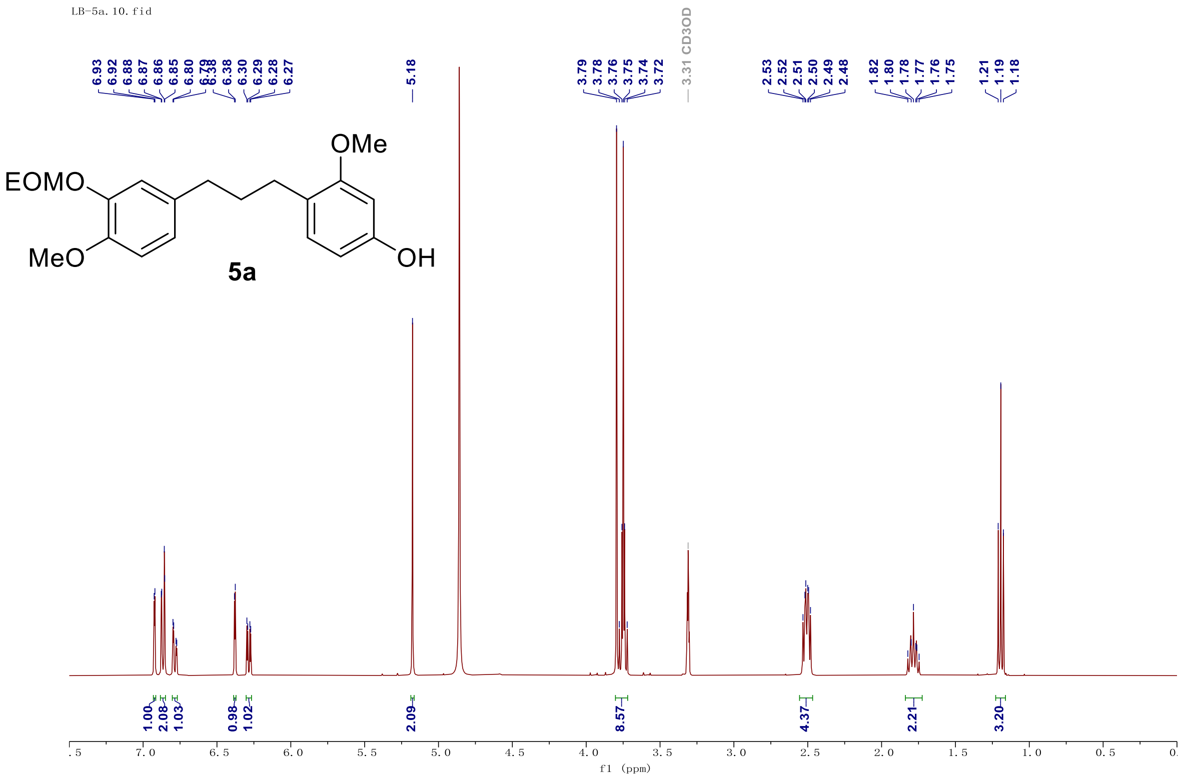

Figure 31. ^{1}H NMR (400 MHz, CD_3OD) spectrum of compound **5a**

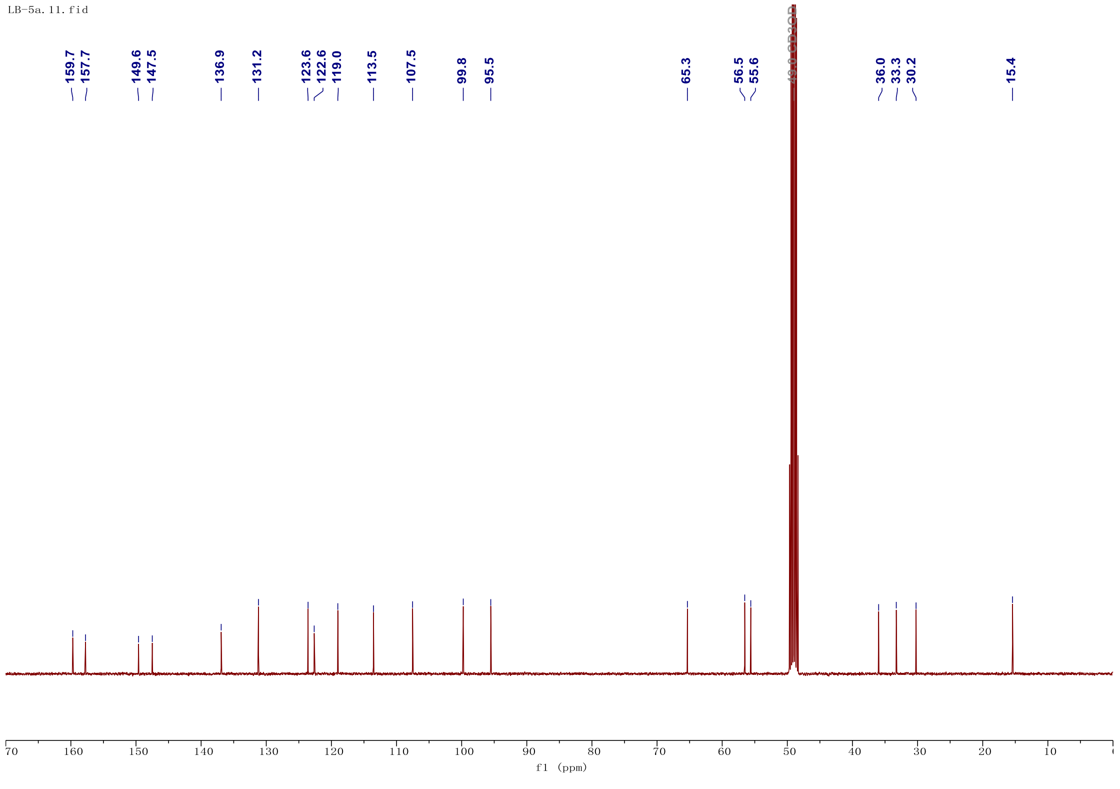

Figure 32. ^{13}C NMR (100 MHz, CD_3OD) spectrum of compound **5a**

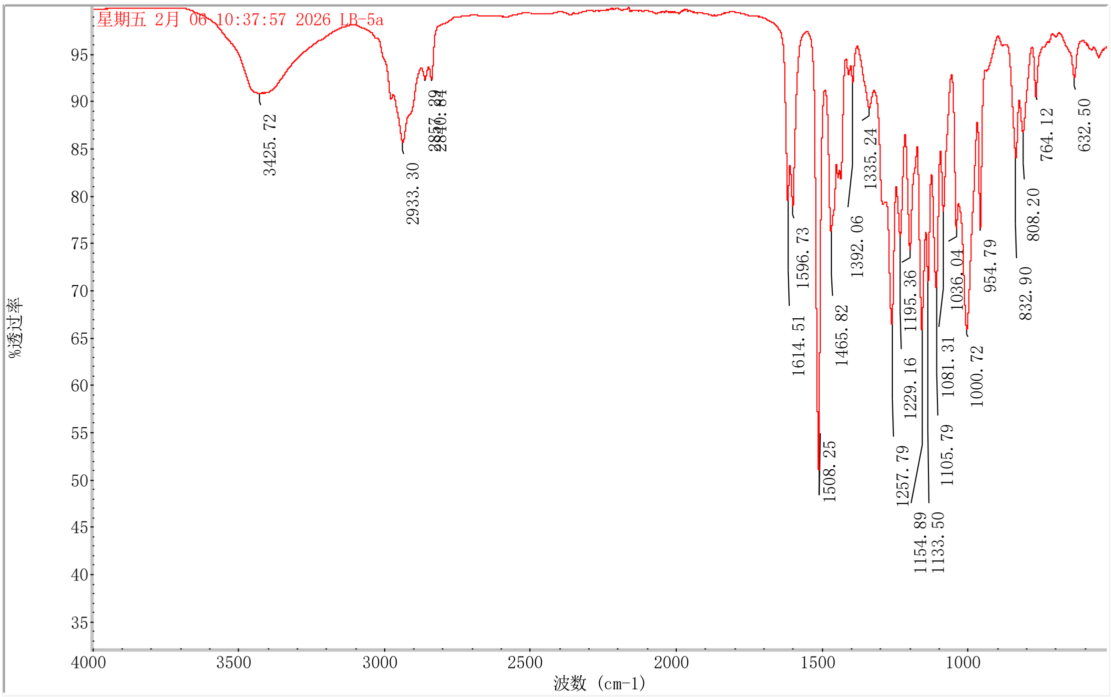

Figure 33. IR spectrum of compound **5a**

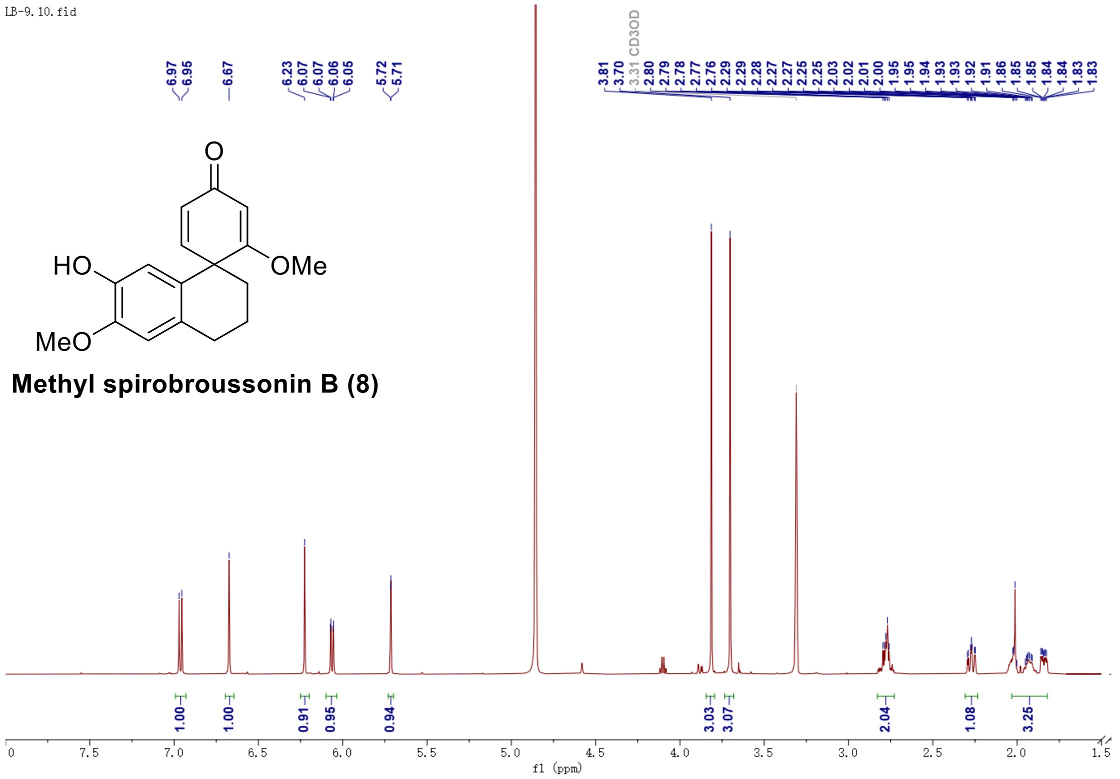

Figure 34. ^{1}H NMR (600 MHz, CD_3OD) spectrum of compound **8**

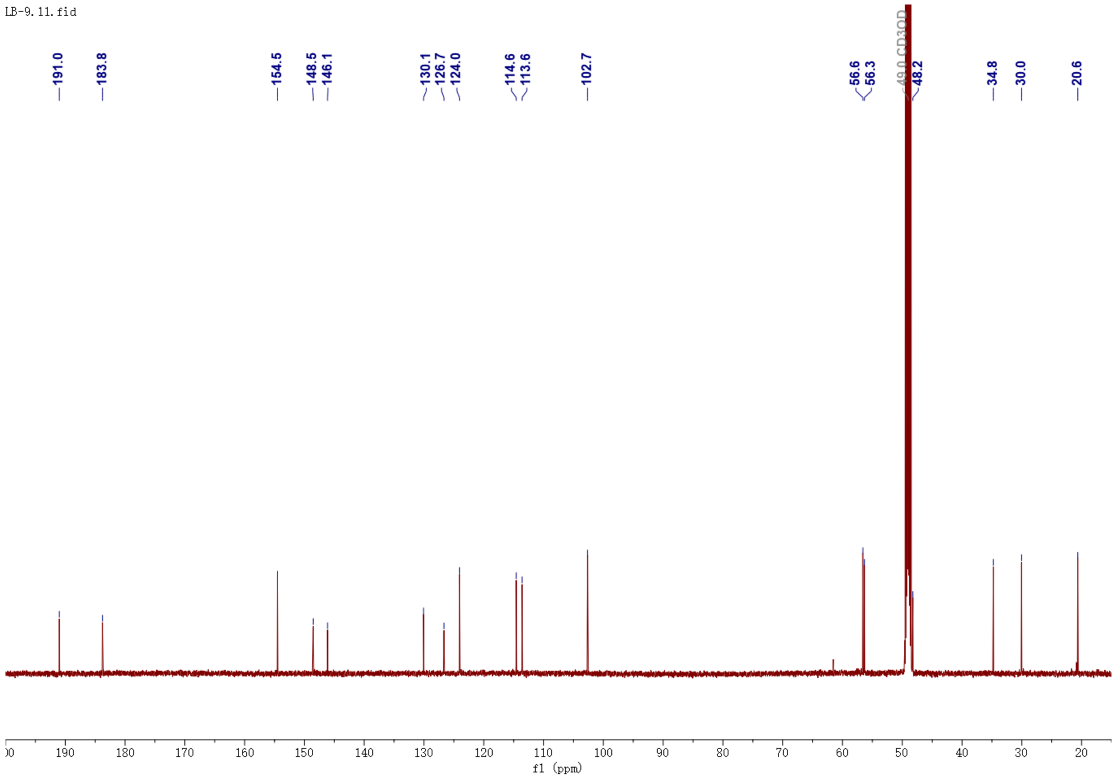

Figure 35. ^{13}C NMR (150 MHz, CD_3OD) spectrum of compound **8**

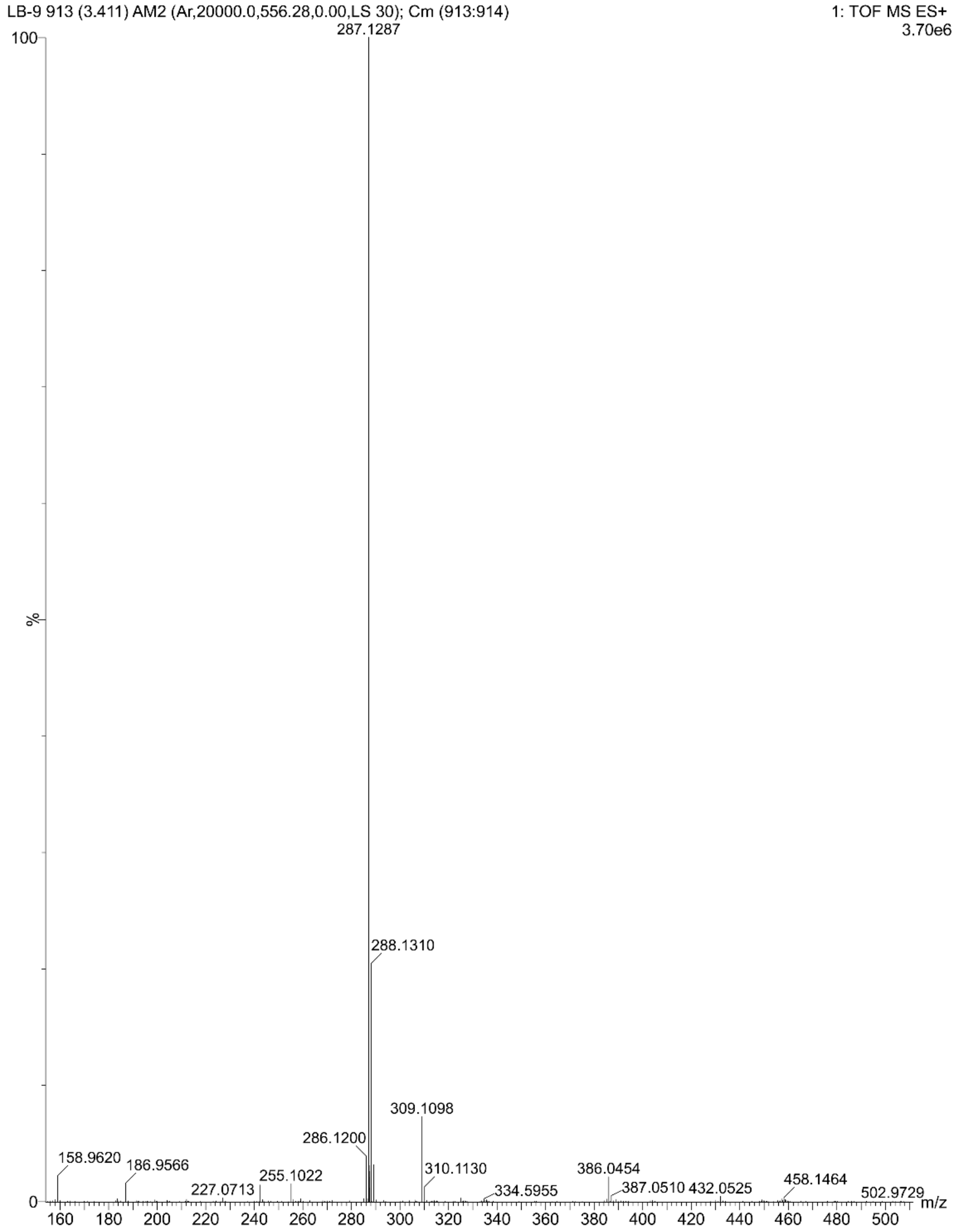

Figure 36. HRMS spectrum of compound **8**
